# An Improved High Yield Total Synthesis and Cytotoxicity Study of the Marine Alkaloid Neoamphimedine: An ATP-Competitive Inhibitor of Topoisomerase IIα and Potent Anticancer Agent

**DOI:** 10.3390/md12094833

**Published:** 2014-09-19

**Authors:** Linfeng Li, Adedoyin D. Abraham, Qiong Zhou, Hadi Ali, Jeremy V. O’Brien, Brayden D. Hamill, John J. Arcaroli, Wells A. Messersmith, Daniel V. LaBarbera

**Affiliations:** 1 Department of Pharmaceutical Sciences, Skaggs School of Pharmacy and Pharmaceutical Sciences, The University of Colorado Anschutz Medical Campus, Aurora, CO 80045, USA; E-Mails: Linfeng.2.Li@ucdenver.edu (L.L.); Adedoyin.Abraham@ucdenver.edu (A.D.A.); Qiong.Zhou@ucdenver.edu (Q.Z.); Hadi.Ali@ucdenver.edu (H.A.); Jeremy.OBrien@ucdenver.edu (J.V.O.); Brayden.Hamill@ucdenver.edu (B.D.H.); 2 Division of Medical Oncology, School of Medicine, The University of Colorado Anschutz Medical Campus, Aurora, CO 80045, USA; E-Mails: John.Arcaroli@ucdenver.edu (J.J.A.); Wells.Messersmith@ucdenver.edu (W.A.M.)

**Keywords:** marine natural product, pyridoacridine, neoamphimedine, total synthesis, topoisomerase II, cytotoxicity, colorectal cancer, cancer therapeutics

## Abstract

Recently, we characterized neoamphimedine (neo) as an ATP-competitive inhibitor of the ATPase domain of human Topoisomerase IIα. Thus far, neo is the only pyridoacridine with this mechanism of action. One limiting factor in the development of neo as a therapeutic agent has been access to sufficient amounts of material for biological testing. Although there are two reported syntheses of neo, both require 12 steps with low overall yields (≤6%). In this article, we report an improved total synthesis of neo achieved in 10 steps with a 25% overall yield. In addition, we report an expanded cytotoxicity study using a panel of human cancer cell lines, including: breast, colorectal, lung, and leukemia. Neo displays potent cytotoxicity (nM IC_50_ values) in all, with significant potency against colorectal cancer (lowest IC_50_ = 6 nM). We show that neo is cytotoxic not cytostatic, and that neo exerts cytotoxicity by inducing G2-M cell cycle arrest and apoptosis.

## 1. Introduction

Topoisomerase II is a ubiquitous enzyme that is evolutionarily conserved in eukaryotes and essential for modulating the topology of DNA chromatin [1,2,3,4]. In vertebrates, topoisomerase II exists as alpha (TopoIIα) and beta isoforms that display differences in expression and sub-cellular localization with TopoIIα playing a critical role in cell proliferation [4,5,6,7]. In particular, TopoIIα is essential for the relaxation and catenation/decatenation (linking/unlinking) of chromatin DNA [8]. Moreover, TopoIIα dependent decatenation must occur before M phase chromosome segregation in order to prevent mitotic catastrophe [9,10,11]. Consequently, TopoIIα is an important molecular target linked to tumor proliferation and progression in several types of human cancer [12,13]. 

**Figure 1:**
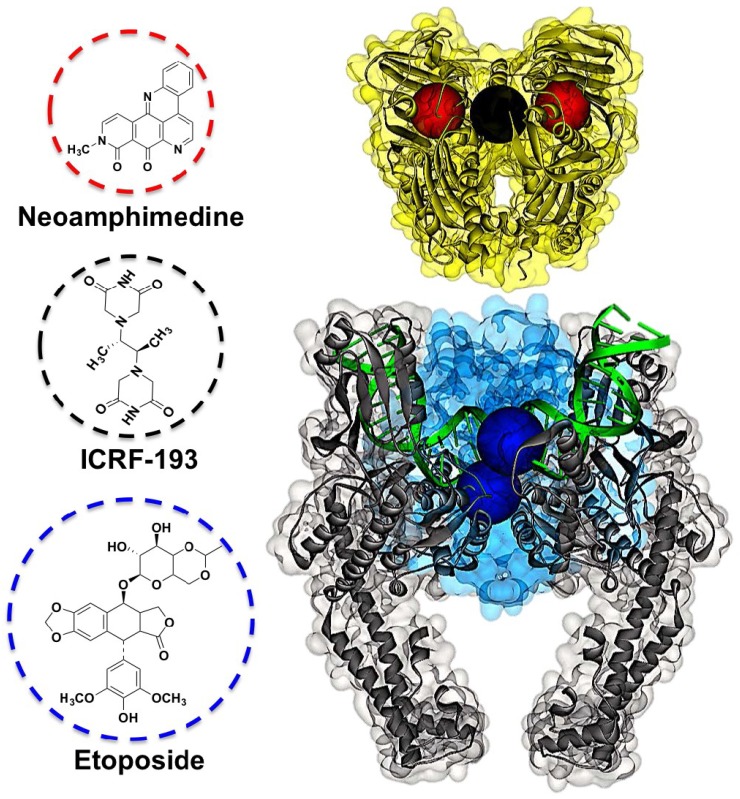
The structures of neoamphimedine (neo) and human TopoIIα homodimer. The *N*-terminal ATPase domain (yellow), neo/ATP binding sites (red spheres), allosteric binding site (black sphere), *C*-terminal DNA cleavage domain (blue) with TopoIIα poison binding sites (dark blue spheres), cleaved DNA (green), and scaffolding domain (grey). The structures of TopoIIα inhibitors are shown with dashed coordinated colored circles indicating their respective binding sites. The TopoIIα structure is a composite of crystal structures using Discovery Studio (Accelrys, San Diego, CA, USA), including: The *N*-terminus (PDB: 1ZXM), and the *C* terminus dimer generated by PISA software using the monomer (PDB: 4FM9).

Human TopoIIα is a homodimer comprised of 1531 amino acids containing three domains: The *N*-terminus ATPase domain and the *C*-terminus scaffold domain, which are connected via DNA binding and cleavage domains also located in the *C*-terminus [3,4] (Figure 1). Conventional TopoIIα drugs (e.g., etoposide) bind to the *C*-terminus and intercalate the DNA cleavage domain [14]. This binding mode stabilizes a transient cleaved complex with DNA, referred to as TopoIIα poisoning. Accumulation of this complex results in DNA damage leading to non-specific cytotoxicity and antitumor activity. While TopoIIα poisons have proven to be effective anticancer drugs they are plagued by drug resistance and severe adverse effects, including chromosomal translocations and secondary malignancies [8,15]. Thus, catalytic TopoIIα inhibitors that do not stabilize the cleaved complex have been seriously pursued [13,16,17]. Arguably, the most successful catalytic inhibitors for the treatment of cancer are the bisdioxopiperazines (e.g., ICRF-193 shown in Figure 1) [18]. 

These drugs display modest anti-tumor activity and are primarily used for reducing cardiotoxicity induced by doxorubicin generated reactive oxygen species (ROS) [19]. Interestingly, their cardioprotective effects are not due to inhibiting TopoIIα, rather it is due to their ability to chelate iron [20,21]. When TopoIIα and DNA are in a closed clamp complex bound to ATP the bisdioxopiperazines bind allosterically stabilizing the clamp [22]. These drugs act as TopoIIα poisons and like the cleaved complex, accumulation of the closed clamp has been reported to cause DNA damage and therefore may also potentiate adverse side effects [8,23]. Therefore, there is still an unmet need to identify effective catalytic inhibitors that do not cause significant non-specific DNA damage. Marine organisms prove to be a rich source of natural products that display catalytic inhibition of TopoIIα [24]. Nevertheless, the development of these agents has been limited, in part, due to a poor understanding and characterization of the exact mode by which these agents bind and catalytically inhibit TopoIIα. 

In our pursuit of novel TopoIIα catalytic inhibitors we have been developing the marine alkaloid neoamphimedine (neo) (Figure 1). Recently, we characterized neo as an ATP-competitive inhibitor of the *N*-terminus of TopoIIα [25]. Thus, far, neo is the only member of the pyridoacridine family with this specific mechanism of action. 

We and others have shown neo to be a potent cytotoxic agent using various human tumor cell lines [25,26,27,28,29,30]. Furthermore, neo displays effective *in vivo* antitumor activity by inhibiting the growth of HCT-116 and KB xenograft tumors in mice [29]. Importantly, neo does not stabilize TopoIIα-DNA complexes [29], does not cause DNA strand breaks or readily intercalate DNA below 100 μM concentrations, and does not induce reactive oxygen species (ROS) [31]. Interestingly, neo is a potent cytotoxic agent in cells overexpressing the multi-drug resistant pump P-glycoprotein (Pgp) [29], indicating that it is not a substrate for efflux drug resistance. We have shown that neo overcomes drug resistance, observed with TopoIIα poisons, due to protein-protein interactions that occur via *C*-terminus interactions [25]. Thus, we hypothesize that ATP-competitive inhibition of TopoIIα may be a more effective way to target TopoIIα with limited DNA damage and multidrug resistance. Currently, we are intensely investigating the *in vitro* anticancer pharmacology and *in vivo* antitumor efficacy of neo. However, our progress has been hindered due to a lack of supply of neo. Although we have reported the first total synthesis of neo [32], which was followed by a slightly different version from Kubo and co-workers [33], the respective overall yields are only 2% and 6%. Herein we report an improved synthesis of neo with an overall yield of 25% and novel biological studies not previously reported. We show that neo produced by this method displays potent cytotoxicity in a panel of human cancers, including: breast, colorectal, lung, and Leukemia. We have characterized neo’s mechanism of cytotoxicity as G2-M cell cycle arrest and apoptosis. 

## 2. Results and Discussion

### 2.1. An Improved Total Synthesis of Neoamphimedine

**Figure 2:**
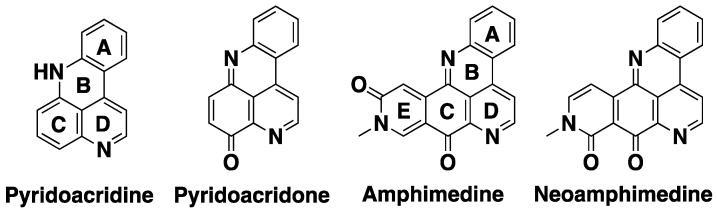
The acridine/acridone skeleton and structures of amphimedine and neoamphimedine.

Neo belongs to the family of compounds known as the pyridoacridines (Figure 2), which consists of more than one hundred natural and synthetic derivatives that display a range of biological activities [26,28,32,34,35]. As a result, the total syntheses of active pyridoacridines have been well studied over the past thirty years [26,34,36,37]. Many of these have been reported in the literature as catalytic inhibitors of TopoIIα [24]. Molinski first reported neo’s structure and activity in a 1993 review and then later by Ireland’s group in 1999 [27,34]. Subsequent to these initial reports, Barrows and Marshall reported neo’s broad antitumor activity *in vitro* and *in vivo* [29]. Arguably, neo is one of the most potent antitumor agents of the pyridoacridine family [28]. Yet, neo was not synthesized until 2007 [32], which is attributed to a much more challenging synthesis evidenced by relatively more steps and lower overall yields compared to other pyridoacridines. For example, Echavarren and Stille first synthesized amphimedine (Figure 2), the parent derivative of neo that differs by one functional group, in 1988 with an overall yield of 23% in eight steps [38]. Subsequent reports by Kubo [39,40], Guillier *et al*. [41], Prager *et al*. [42] and Bracher and Papke [43] followed. In all of these cases, the synthesis of amphimedine was accomplished using palladium catalyzed crossed coupling and/or hetero Diels-Alder reactions to form the **E**-ring (Figure 2). While palladium catalyzed cross coupling can be used to generate the **A** and **B** rings of neo, the formation of the **E**-ring is not possible via the Diels-Alder reaction. Instead, LaBarbera and Ireland prepared neo’s **E**-ring by first introducing a carboxcylic acid in the seven position (numbering based on the quinoline ring) via a Sandmeyer reaction with CuCN and subsequent hydrolysis, followed by amide formation with methylamino dimethyl acetal and acid catalyzed ring closure [32]. Recently, Kubo and co-workers attempted to improve the neo synthesis by generating the **E**-ring using Bischler-Napieralski cyclization from a phenethyl amine functionality [33]. Both of these syntheses required 12 steps and resulted in low overall yields as described above. 

We present here a redesigned neo synthesis that is more efficient and higher yielding. Overall, the design of this improved synthesis was inspired by the work described above, particularly from the original neo synthesis by LaBarbera *et al*. [32] and subsequent synthesis by Nakahara *et al*. [33]. Our initial design and plan examined the formation of the **E**-ring early on in the synthesis using both the acid catalyzed ring closure reported by LaBarbera and the Curtius thermal rearrangement a cyclization, which is a well-known method to generate isoquinolone ring systems [44,45]. 

**Scheme 1:**
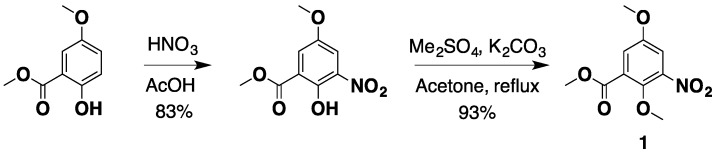
The synthesis of nitro starting material **1**.

All of these methods failed to provide the desired **E**-ring, which we thoroughly describe in the Supplementary Information. Nevertheless, these tribulations led us to the design and successful completion of the high yielding and efficient synthesis of neo (25% overall yield in 10 steps). The synthesis begins from commercially available methyl 2,5-dimethoxy-3-nitrobenzoate (**1**). Alternatively, due to the relatively high cost of **1**, it can be synthesized in 2 steps, in 77% yield from methyl 5-methoxysalicylate using a modified reported method [46] (Scheme 1). 

**Scheme 2:**
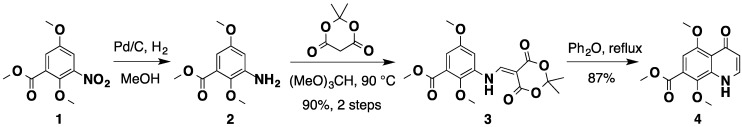
The synthesis of the quinolone intermediate **4**.

In our previous synthesis of neo, the **A** and **D** rings (Figure 2), were prepared in four steps via Knorr cyclization. However, this method was cumbersome requiring viscous polyphosphoric acid and in general gave poor yields. Therefore, to prepare the **A** and **D** rings we utilized the Meldrum’s derivatization and subsequent palladium catalyzed cross coupling reaction, which was reported by Stille [38] and Kubo [33] to be efficient and high yielding. Catalytic hydrogenation of the nitrobenzoate **1** gave aniline **2**, which was transformed into compound **3** by treatment with Meldrum’s acid and trimethyl orthoformate. Subsequent thermal ring closure afforded the key quinolone intermediate **4** with an overall yield of 78% in three steps without flash chromatography (Scheme 2).

**Scheme 3:**
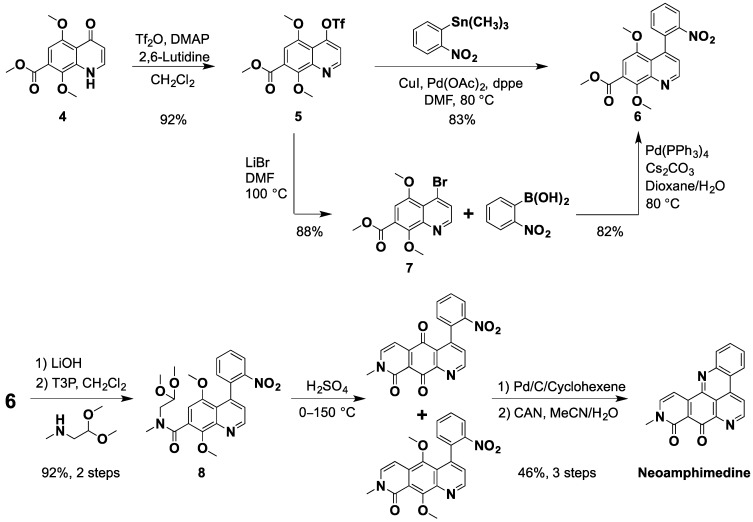
The synthesis neoamphimedine from quinolone **4**.

Next, quinolone **4** was then converted to the triflate ester by stirring in dry dichloromethane in the presence of trifluoromethanesulfonic anhydride using catalytic amounts of dimethyl amino pyridine (DMAP) [38], producing **5** in 92% yield. Palladium catalyzed Stille coupling of **5** with the readily available trimethyl (2-nitrophenyl) stannane [47] afforded **6** in 83% yield. Similarly, this key nitro intermediate can also be prepared by the Suzuki coupling of bromide **7** and 2-nitrophenyl boronic acid [33,48] (Scheme 3). First, we attempted Suzuki coupling with the triflate **5**, which failed giving only the parent quinolone **4** due to hydrolysis. Next, we tried direct conversion of **4** to **7** using POBr_3_ or PBr_3_ but this method was abandoned due to very low yields. However, reaction of triflate **5** with LiBr in DMF provided bromide **7** smoothly. While the direct Stille coupling of **5** gave **6** in 83% yield in one step, we elected to scale up using the 2-step bromination-Suzuki sequence from **5**, in 72% yield, due to the relatively higher cost and known toxicity of organotin compounds [49].

The ester **6** was hydrolyzed using lithium hydroxide and the corresponding acid was then coupled to methylamino acetaldehyde dimethylacetal with “green” reagent propylphosphonic anhydride (T3P) to give amide **8** in 92% yield over two steps. The **E**-ring of neo was formed using our previously reported acid catalyzed ring closure method [32], which provides a mixture of dimethoxy and quinone, intermediates. This mixture was catalytically reduced followed by oxidative demethylation with ceric ammonium (IV) nitrate (CAN) to give neo as an orange solid in 46% yield. All spectral data were in accordance with previously reported values for neo [27,32]. In summary, the improved synthesis of neo was achieved in 10 steps with an overall yield of 25%, which is 4.2 fold better than Nakahara *et al*. [33] and 12.5 fold better than LaBarbera *et al*. [32]. 

**Table 1:** Cytotoxicity IC_50_ values obtained of neo compared to etoposide. The etoposide values are taken as reported in reference ** [50] or obtained from * the CancerDR: Drug resistance data base [51].

Human Cancer Cells	Neo IC_50_ (μM)	Etoposide IC_50_ (μM)
**Breast**		
MCF7	0.433	0.83 *
MDA-MB-231	0.76	9.03 *
PMC42LA	0.723	—
T47D	0.740	11.58 *
**Colorectal**		
HCT116	0.229	1.01 *
SW48	0.006	0.87 *
SW480	0.383	6.4 **
SW620	0.060	0.39 *
**Leukemia**		
HEL	0.135	3.88 *
Kasumi I	0.244	1.32 *
Molm13	0.018	0.38 *
OCI AML3	0.036	—
**Lung**		
A549	0.489	3.9 **
H2009	1.095	—
HCC827	2.957	—
SW1573	0.157	2.25 *

### 2.2. Cytotoxicity Studies with Neo Using a Panel of Human Colorectal Cancer Cell Lines

As described above, neo proves to be cytotoxic against various human cancer cells [25,26,27,28,29,30]. To further characterize neo’s anticancer activity we conducted sulforhodamine B (SRB) assays for adherent cells [52] and the acid phosphatase assay for non-adherent cells [53] using a panel of human cancer cell lines (Table 1). As expected, neo displayed potent cytotoxicity in all cell lines tested. In particular, neo was highly cytotoxic against colorectal cancer cells with IC_50_ values ranging between 6 nM to 383 nM. The most potent activity was observed against SW48 (6 nM) as well as the metastatic SW620 cells (60 nM) [54]. Furthermore, neo was significantly more cytotoxic than the conventional TopoIIα poison, etoposide. Interestingly, TopoIIα poisons are not prescribed for the treatment of colorectal cancers, presumably due to poor response and drug resistance observed in the clinic [55].

While neo appears to be cytotoxic, we utilized the SRB recovery assay to determine whether the inhibitory effect of neo is indeed cytotoxic or potentially cytostatic in nature [56,57]. Thus, the SRB recovery assay was performed simultaneously using HCT116, SW620, and SW480 human colorectal cancer cell lines. If neo has a cytostatic effect, the cells will begin to divide during the 72 h recovery time following the removal of neo, and the overall growth in the treatment wells will be similar to cell growth in control wells. If neo is cytotoxic, there will be little difference in cell growth between the SRB plate and the SRB recovery plate, and the IC_50_ values should be comparable. Our results exhibited no change in cell growth during the 72 h recovery period and displayed virtually identical IC_50_ values compared to the parallel SRB assay, illustrating that neo is cytotoxic in nature.

**Figure 3:**
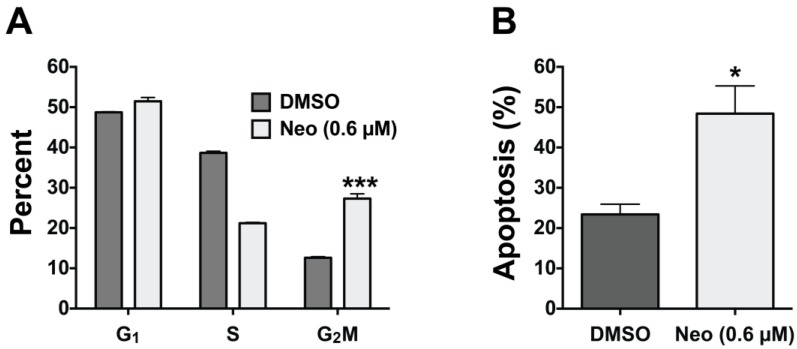
(**a**) FACS cell cycle analysis of SW48 cells treated with neo for 48 h. The bar graph indicates the different phases of the cell cycle showing statistically significant increase in the G2-M phase, indicative of a cell cycle arrest mechanism; (**b**) FACS analysis quantifying annexin V-positive staining of SW48 cells treated with neo over 48 h indicating apoptosis. *t*-test analysis indicates significance where * *p* ≤ 0.05 or *** *p* ≤ 0.001.

### 2.3. Neo Exerts Cytotoxicity by Inducing G2-M Cell Cycle Arrest and Apoptosis

Although neo has been shown to be cytotoxic the exact mechanism of cell death has not been characterized. Therefore, using fluorescence-activated cell sorter (FACS) analysis [58,59], we measured neo’s effect on the cell cycle and apoptosis using SW48 cells treated with 0.60 μM neo for 48 h (Figure 3). Propidium iodide staining followed by FACS indicated that the percentage of cells in the G1 phase of the cell cycle were the same for both neo treated and control cells. However, we observed a decrease in the S phase population and a statistically significant increase in the G2-M phase population (Figure 3A). Taken together, these data indicate that neo induces cell cycle arrest in the G2-M phase of the cell cycle. Interestingly, TopoIIα dependent chromatin decatenation has been shown to be required for G2-M phase cell cycle progression and inhibiting TopoIIα dependent decatenation arrests cells in the G2-M phase [11]. However, TopoIIα poison (e.g., etoposide) induced DNA damage does not recapitulate this cell cycle arrest mechanism [60]. Thus, the data shown in Figure 3 support our previous finding that neo acts via ATP-competitive inhibition of the *N*-terminal ATPase domain of TopoIIα but not as a TopoIIα poison that results in significant DNA damage. Our data are also in agreement with findings by Barrows and co-workers who have shown that neo does not induce significant DNA damage or intercalate DNA below 100 μM [29,31]. Next, using propidium iodide co-stained with annexin V, we measured neo’s ability to induce apoptosis. Annexin V staining detects exposed phosphatidyl serine, which is an early event and biomarker of apoptosis [61]. FACS analysis showed that neo induced a statistically significant two-fold induction of annexin V staining compared to the vehicle control cells, indicating that neo induces cell death by apoptosis.

## 3. Experimental Section

### 3.1. General Experimental Procedures

All reagents were purchased from commercial sources and used as received, unless otherwise indicated. All solvents were dried and distilled using standard protocols. All reactions were carried out under a nitrogen atmosphere unless otherwise noted. All organic extracts were dried over sodium sulfate. Thin layer chromatography (TLC) was performed using aluminum-backed plates coated with 60Å Silica gel F254 (Sorbent Technologies, Norcross, GA, USA). Plates were visualized using a UV lamp (λ_max_ = 254 nm) and/or by staining with phosphomolybdic acid solution (20 wt% in ethanol). Column chromatography was carried out using 230–400 mesh 60Å silica Gel. Proton (δ_H_) and carbon (δ_C_) nuclear magnetic resonances were recorded on a Varian INOVA 500 MHz spectrometer (500 MHz proton, 125.7 MHz carbon, Agilent Technologies, Santa Clara, CA, USA). High-resolution mass spectra (HRMS) were recorded on a Bruker Q-TOF-2 Micromass spectrometer (Bruker, Fremont, CA, USA) equipped with lock spray, using ESI with methanol as the carrier solvent. Accurate mass measurements were performed using leucine enkephalin as a lock mass and the data were processed using MassLynx 4.1 (Bruker, Fremont, CA, USA). Exact *m/z* values are reported in Daltons. Melting points (m.p.) were determined using a Stuart SMP40 melting point apparatus (Bibby Scientific, Staffordshire, UK) and are uncorrected. Infrared (IR) spectra were recorded on a Bruker ALPHA FT-IR fitted (Bruker, Bellerica, MA, USA) with a Platium ATR diamond sampler (oils and solids were examined neat). Absorption maxima (ν_max_) are recorded in wavenumbers (cm^−1^). The ^1^H NMR and ^13^C NMR spectra for all synthetic compounds are provided in the Supplementary Information.

### 3.2. Cell Culture

Human breast, colorectal, lung, and leukemia cancer cell lines were cultured using Hyclone media containing 5% FBS (Hyclone, Life Technologies, Grand Island, NY, USA) and maintained under standard humidified incubation at 37 °C in 5% CO_2_, including: MEM (T47D), DMEM (MCF7), or RPMI (MDA-MB-231, PMC42LA, HCT116, SW48, SW480, SW620, A549, H2009, HCC827, SW1573, HEL, Kasumi 1, Molm13, OCI AML3). 

### 3.3. Sulforhodamine B (SRB) Cytotoxicity Assay

Breast, colon, and lung cancer cells (4000/well for PMC42LA; 3000/well for MCF7 and MDA-MB-231; 2500/well for SW48 and SW480; 2000 for SW620; 1500 for H2009, HCC827, SW1573, and T47D; and 1000/well for A549 and HCT116) were plated in triplicate in 96-well plates (100 μL/well). One well containing media only was included as the background control. After 24 h, cells were treated with increasing doses of neo (100 μL/well). Cell growth was analyzed using the SRB assay [52]. Briefly, after 72 h of drug exposure, cells were fixed with 10% trichloroacetic acid (Sigma T6399, St. Louis, MO, USA) at 4 °C for 30 min, washed with double distilled water (ddH_2_O), and stained with 0.057% SRB (Sigma S1402, St. Louis, MO, USA). Unbound stain was removed by washing with 1% acetic acid. Protein-bound SRB was solubilized with 10 mmol/L unbuffered Tris base, and the optical density was measured at an absorbance wavelength of 570 nm. 

### 3.4. SRB Recovery Assay

The SRB cytotoxicity assay was followed through 72 h of drug exposure as described in Section 3.3. At the end of the 72 h neo treatment, the growth medium was removed and the remaining cells were washed once with 200 μL of sterile PBS. The PBS was replaced with 200 μL of complete growth medium and the cells were allowed to recover for 72 h. After recovery, cells were fixed with 10% trichloroacetic acid (Sigma T6399, St. Louis, MO, USA) at 4 °C for 30 min, washed with double ddH_2_O, and stained with 0.057% SRB (Sigma S1402, St. Louis, MO, USA). Unbound stain was removed by washing with 1% acetic acid. Protein-bound SRB was solubilized with 10 mmol/L unbuffered Tris base, and the optical density was measured at an absorbance wavelength of 570 nm. 

### 3.5. Acid Phosphatase (APH) Assay

Leukemia cells (5000 in 100 μL per well) were plated in triplicate in 96-well plates using phenol red free RPMI (Invitrogen, Life Technologies, Grand Island, NY, USA) containing 5% FBS (Hyclone). One well containing media only was included as the background control. Twenty-four hours later, cells were treated with increasing doses of neo (25 μL/well). Cell growth was analyzed using the APH assay [53]. Briefly, 125 μL of APH assay buffer (0.1 M sodium acetate (pH 5.0), 0.1% Triton X-100, and 10 mM *p*-nitrophenyl phosphate) was added to each well and the plates were incubated at 37 °C for 90 min. The optical density was measured at an absorbance wavelength of 405 nm after adding 10 μL of 1 N NaOH to each well. 

### 3.6. FACS Analysis of Cell Cycle Distribution and Apoptosis

Cell cycle and apoptosis experiments were conducted as previously reported with slight modification [59]. Briefly, SW48 cells (2 × 10^5^ per well) were seeded into six-well plates and incubated for 24 h. Cells were treated with neo (0.6 μM) for 48 h, washed with PBS, and trypsinized with 0.5 mL of 0.25% trypsin. Cells were collected by centrifugation (1000 rpm, 5 min), washed twice with 2 mL of ice-cold 1 × PBS (pH 7.4) and resuspended in staining buffer (1 × Dulbecco’s PBS, 3% FCS, 0.09% NaN_3_, pH 7.4) containing 5 μg of propidium iodide (Sigma P-4170, St. Louis, MO, USA). The cells were stained for 24 h at 4 °C in the dark followed by FACS cell cycle analysis. Likewise, SW48 cells treated with neo were also assessed for apoptosis using an annexin V Apoptosis Detection kit (eBioscience-cat# BMS500FI, San Diego, MO, USA) according to manufacture instructions. Cells were resuspended in 200 μL binding buffer and treated with 5 μL of annexin V-FITC, incubated for 10 min at room temperature in the dark and then washed with 200 μL binding buffer. Next, 10 μL of propidium iodide (20 μg/mL) was added followed by FACS analysis. FACS analyses were carried out at the University of Colorado Cancer Center Flow Cytometry core facility using a Beckman Coulter Gallios 561 flow cytometer (Beckman Coulter, Brea, CA, USA) equipped with Kaluza G acquisition software (Beckman Coulter, Brea, CA, USA).

### 3.7. Synthetic Procedures

#### 3.7.1. Methyl 2-hydroxy-5-methoxy-3-nitrobenzoate

A solution of methyl 2-hydroxy-5-methoxybenzoate (5.70 g, 31.3 mmol) in acetic acid (33 mL) was stirred between 15 and 20 °C. A solution of HNO_3_ (2.40 mL, 37.5 mmol) in acetic acid (9 mL) was slowly added while maintaining the reaction temperature below 20 °C. The reaction mixture was stirred at room temperature for 1 h, 100 mL of ice water was then added. The solid was filtered off and dried to give the nitrated compound (5.90 g, 26.0 mmol, 83%) as an orange solid. TLC (35% ethyl acetate in hexane) R*_f_* = 0.40. Spectral analysis were in accordance with previously reported values [62].

#### 3.7.2. Methyl 2,5-Dimethoxy-3-nitrobenzoate (**1**)

To a stirred suspension of methyl 2-hydroxy-5-methoxy-3-nitrobenzoate (6.94 g, 30.5 mmol) and anhydrous K_2_CO_3_ (6.33 g, 45.8 mmol) in acetone (40 mL) was added dimethyl sulfate (3.48 mL, 36.6 mmol) drop wise. The reaction mixture was heated to reflux for 5 h and then filtered. The filtrate was concentrated and recrystallized with methanol to afford **1** (6.88 g, 28.5 mmol, 93%) as a white crystlline solid. TLC (35% ethyl acetate in hexane) R*_f_* = 0.48. All spectral analysis results were in accordance with previously reported values [46].

#### 3.7.3. Methyl 3-((2,2-Dimethyl-4,6-dioxo-1,3-dioxan-5-ylidene)methyl-amino)-2,5-dimethoxy-benzoate (**3**)

Ester **1** (2.86 g, 11.86 mmol) and palladium on carbon (10 wt% loading, 126.2 mg, 0.119 mmol) in MeOH (20 mL) were shaken in a Parr hydrogenator for 3 h under 50 psi of H_2_. After filtration of the catalyst, evaporation of the filtrate under reduced pressure gave the amine **2** as an orange oil, which was used without further purification. A solution of 2,2-dimethyl-1,3-dioxane-4,6-dione (2.57 g, 17.8 mmol) in trimethyl orthoformate (25 mL) was heated at 90 °C for 2 h and the crude amine **2** was added, the reaction mixture was stirred at 90 °C for another 2 h. After the reaction mixture was cooled to room temperature it was filtered through silica gel and recrystallized from methanol to afforded **3** (3.91 g, 10.7 mmol, 90%) as a yellow solid. M.p. 132–134 °C; IR (neat) ν_max_ 3234, 2982, 1713, 1678, 1627, 1598, 1455, 1260 cm^−1^; TLC (35% ethyl acetate in hexane) R*_f_* = 0.30; ^1^H NMR (500 MHz, CDCl_3_) δ 11.73 (d, *J* = 14.0 Hz, 1H), 8.66 (d, *J* = 14.5 Hz, 1H), 7.19 (d, *J* = 3.0 Hz, 1H), 7.10 (d, *J* = 3.0 Hz, 1H), 3.96 (s, 3H), 3.92 (s, 3H), 3.86 (s, 3H), 1.76 (s, 6H); ^13^C NMR (125.7 MHz, CDCl_3_) δ 165.1, 165.0, 163.3, 155.9, 150.8, 143.7, 133.1, 125.8, 112.8, 105.3, 105.0, 88.2, 62.7, 55.9, 52.5, 27.0; ESI-HRMS calcd. for C_17_H_19_NO_8_Na [M + Na]^+^ 388.1003, found 388.1011.

#### 3.7.4. Methyl 1,4-Dihydro-5,8-dimethoxy-4-oxo-quinoline-7-carboxylate (**4**)

The meldrum derivative **3** (1.38 g, 3.78 mmol) in diphenyl ether (8 mL) was refluxed under nitrogen for 30 min. After the mixture was cooled to room temperature, hexane (100 mL) was added. Filtration of the reaction mixture afforded the quinolone **4** (868.3 mg, 3.30 mmol, 87%) as a yellow solid. M.p. 233 °C; IR (neat) ν_max_ 3071, 2996, 2943, 1709, 1609, 1580, 1508, 1224 cm^−1^; TLC (10% MeOH in ethyl acetate) R*_f_* = 0.10; ^1^H NMR (500 MHz, DMSO-*d*_6_) δ 11.21 (s, 1H), 7.68 (d, *J* = 6.5 Hz, 1H), 6.89 (s, 1H), 5.98 (d, *J* = 7.5 Hz, 1H), 3.90 (s, 3H), 3.82 (s, 3H), 3.79 (s, 3H); ^13^C NMR (125.7 MHz, DMSO-*d*_6_) δ 176.5, 165.2, 154.8, 142.2, 137.9, 136.9, 124.5, 119.0, 112.5, 103.0, 62.7, 55.9, 52.6; ESI-HRMS calcd. for C_13_H_13_NO_5_Na [M + Na]^+^ 286.0686, found 286.0675.

#### 3.7.5. Methyl 5,8-Dimethoxy-4-trifluoromethanesulfonyloxy-quinoline-7-carboxylate (**5**)

To a stirred solution of quinolinone **4** (1.65 g, 5.85 mmol), 2,6-dimethylpyridine (1.02 mL, 8.77 mmol) and DMAP (142.9 mg, 1.17 mmol) in CH_2_Cl_2_ (12 mL) at 0 °C was added trifluoromethanesulfonic anhydride (1.04 mL, 6.14 mmol) drop wise. The reaction mixture was allowed to warm up to room temperature, stirred for 4 h and then concentrated. Chromatographic purification on silica gel (35% ethyl acetate in hexane) afforded triflate **5** (2.13 g, 5.39 mmol, 92%). Yellow solid; M.p. >86 °C (dec); IR (neat) ν_max_ 2949, 1698, 1606, 1424, 1381, 1202 cm^−1^; TLC (50% ethyl acetate in hexane) R*_f_* = 0.49; ^1^H NMR (500 MHz, CDCl_3_) δ 9.03 (d, *J* = 5.0 Hz, 1H), 7.32 (d, *J* = 5.0 Hz, 1H), 7.28 (s, 1H), 4.15 (s, 3H), 4.03 (s, 3H), 4.02 (s, 3H); ^13^C NMR (125.7 MHz, CDCl_3_) δ 166.3, 153.1, 151.0, 150.6, 149.9, 147.1, 125.0, 118.9 (q, ^1^*J*_C–F_ = 320.9 Hz), 117.5, 115.4, 107.0, 63.7, 55.9, 52.9; ESI-HRMS calcd. for C_14_H_13_F_3_NO_7_S [M + H]^+^ 396.0359, found 396.0367.

#### 3.7.6. Methyl 4-Bromo-5,8-dimethoxy-quinoline-7-carboxylate (**7**)

A suspension of triflate **5** (502.1 mg, 1.27 mmol) and LiBr (551.6 mg, 6.35 mmol) in DMF (4 mL) was heated to 80 °C for 1 h. After cooling to room temperature, the mixture was diluted with ethyl acetate and washed with sat. NaHCO_3_. The organic extracts were dried over Na_2_SO_4_, filtered and concentrated in vacuo to give the desired bromide **7** (364.7 mg, 1.12 mmol, 88%). Light yellow solid; M.p. 121–122 °C; IR (neat) ν_max_ 2935, 1731, 1614, 1566, 1459, 1095 cm^−1^; TLC (50% ethyl acetate in hexane) R*_f_* = 0.47; ^1^H NMR (500 MHz, CDCl_3_) δ 8.67 (d, *J* = 4.5 Hz, 1H), 7.77 (d, *J* = 4.5 Hz, 1H), 7.24 (s, 1H), 4.12 (s, 3H), 4.00 (s, 3H), 3.98 (s, 3H); ^13^C NMR (125.7 MHz, CDCl_3_) δ 166.5, 151.1, 150.8, 149.5, 145.9, 129.2, 129.0, 123.9, 122.8, 106.6, 63.5, 55.9, 52.7; ESI-HRMS calcd. for C_13_H_13_BrNO_4_ [M + H]^+^ 326.0023, found 326.0031.

#### 3.7.7. Methyl 5,8-Dimethoxy-4-(2-nitrophenyl)-quinoline-7-carboxylate (**6**)

From triflate **5**: To a stirred solution of triflate **5** (1.29 g, 3.26 mmol), CuI (310.4 mg, 1.63 mmol) and 2-nitrophenyltrimethyl stanne (1.40 g, 4.89 mmol) in DMF (6 mL) were added Pd(OAc)_2_(14.6 mg, 65.2 μmol) and dppe (26.0 mg, 65.2 μmol). The reaction mixture was heated to 75 °C for 6 h. After cooling to room temperature, the dark reaction mixture was filtered through celite and concentrated, chromatographic purification on silica gel (35% ethyl acetate in hexane) provided **6** (990.7 mg, 2.69 mmol, 83%) as a light yellow solid; M.p. 159–160 °C; IR (neat) ν_max_ 2938, 2846, 1726, 1515, 1460, 1373, 1230 cm^−1^; TLC (50% ethyl acetate in hexane) R*_f_* = 0.30; ^1^H NMR (500 MHz, CDCl_3_) δ 9.03 (d, *J* = 4.5 Hz, 1H), 8.20–8.18 (dd, *J* = 1.5, 8.0 Hz, 1H), 7.69–7.65 (dt, *J* = 1.5, 7.5 Hz, 1H), 7.59–7.56 (dt, *J* = 1.5, 7.5 Hz, 1H), 7.31–7.29 (dt, *J* = 1.5, 7.5 Hz, 1H), 7.25 (d, *J* = 4.5 Hz, 1H), 7.06 (s, 1H), 4.20 (s, 3H), 3.99 (s, 3H), 3.47 (s, 3H); ^13^C NMR (125.7 MHz, CDCl_3_) δ 166.7, 151.5, 151.2, 150.0, 147.9, 144.8, 144.4, 137.6, 132.8, 131.0, 128.5, 123.7, 123.3, 123.1, 121.5, 105.5, 63.7, 55.7, 52.6; ESI-HRMS calcd. for C_19_H_17_N_2_O_6_ [M + H]^+^ 369.1081, found 369.1075. From bromide **7**: To a stirred solution of bromide **7** (178.0 mg, 0.546 mmol), Cs_2_CO_3_ (889.4 mg, 2.73 mmol) and 2-nitrophenyl boronic acid (182.3 mg, 1.09 mmol) in dioxane/H_2_O (3 mL, v/v, 10:1) were added Pd(PPh_3_)_4_ (63.1 mg, 54.6 μmol). The reaction mixture was heated to 80 °C for 24 h. After cooling to room temperature, the reaction mixture was filtered through celite and concentrated, chromatographic purification on silica gel (35% ethyl acetate in hexane) provided **6** (167.2 mg, 0.454 mmol, 83%) as a light yellow solid.

#### 3.7.8. 7-[*N*-(2,2-Dimethoxyethyl)-*N*-methyl]-carboxamide-5,8-dimethoxy-4-(2-nitrophenyl) quinoline (**8**)

To a stirred solution of **6** (784.2 mg, 2.12 mmol) in THF/MeOH/H_2_O (7.5 mL, v/v/v, 2:2:1) were added LiOH·H_2_O (268.0 mg, 6.39 mmol) in one portion. The reaction mixture was stirred at room temperature for 10 h and concentrated to yield the crude carboxylic acid. To a stirred solution of carboxylic acid residue and methylamino acetaldehyde dimethylacetal (327 μL, 2.54 mmol) in CH_2_Cl_2_ (6 mL) at 0 °C, was added propylphosphonic anhydride (T3P, 1.87 mL, 50 wt% in ethyl acetate, 3.18 mmol) drop wise. The reaction mixture was allowed to warm up to room temperature and stirred for 3 h. The reaction mixture was diluted with H_2_O (40 mL) and washed with CH_2_Cl_2_ (3 × 30 mL). The combined organic extracts were dried over Na_2_SO_4_, filtered and concentrated to give amide **8** (888.9 mg, 1.95 mmol, 92%) as an orange oil, which was used directly for next step without further purification. All spectral analysis results were in accordance with previously reported values [32].

#### 3.7.9. Neoamphimedine (neo)

Neo was prepared from dimethyl acetal **8** (200.8 mg, 0.441 mmol) as previously reported [32] with minor modifications to the workup. Briefly, after acid catalyzed ring closure (20 min, 150 °C) the reaction was poured into 100 mL of ice water and made basic to pH = 9 with K_2_CO_3_. The aqueous layer was extracted first with ethyl acetate (3 × 100 mL) and then with CHCl_3_ (3 × 100 mL). The ethylacetate extract contains a mixture of dimethoxy (previously reported by LaBarbera *et al*. [32]; TLC, 30% MeOH in ethyl acetate, R*_f_* = 0.41) and we presume hydroquinone (TLC, 30% MeOH in ethyl acetate, R*_f_* = 0.25). In addition, the crude chloroform extract (68.9 mg) contains a mixture of the presumed hydroquinone (TLC, 30% MeOH in ethyl acetate, R*_f_* = 0.25) and quinone (TLC, 50% CHCl_3_ in MeOH, R*_f_* = 0.43). Upon concentration of the organic extracts the presumed hydroquinone rapidly air oxidizes to the quinone, which is stable, thus NMR and MS analysis was possible for the quinone, 8-methyl-4-(2-nitrophenyl)-pyrido-[4,3-*g*]-quinoline-5,9,10-trione: ^1^H NMR (500 MHz, DMSO-*d6*) δ 9.10 (d, *J* = 4.0 Hz, 1H), 8.30 (t, *J* = 6.5 Hz, 2H), 7.87 (t, *J* = 6.5 Hz, 1H), 7.70 (t, *J* = 6.0 Hz, 1H), 7.67 (d, *J* = 4.0 Hz, 1H), 7.43 (d, *J* = 6.0 Hz, 1H), 6.58 (d, *J* = 6.0 Hz, 1H), 3.56 (s, 3H); ^13^C NMR (125.7 MHz, DMSO-*d6*) δ 183.6, 178.0, 157.8, 154.4, 149.2, 147.6, 147.4, 146.6, 144.3, 134.3, 130.6, 129.6, 128.0, 125.5, 124.3, 118.6, 99.5, 38.2; ESI-HRMS calcd. for C_19_H_11_N_3_O_5_Na [M + Na]^+^ 384.0596, found 384.0579. The crude ethyl acetate extract was purified by column chromatography using alumina (Brockman acitivity I neutral, 50% acetone in ethyl acetate to 50% CHCl_3_ in MeOH) to afford a yellow oil (58.7 mg) containing quinone (resulting from hydroquinone air oxidation) and dimethoxy intermediates (see Scheme 3). The mixtures were combined and used without purification. 

Next, to a stirred solution of the combined crude residues in cyclohexene/EtOH (18 mL, v/v, 1:2) was added palladium (10 wt% on carbon, 234.7 mg, 0.221 mmol) and heated at reflux for 90 min and then filtered using a fine fritted glass filter. Note: the reduced mixture is very sticky and no celite or filter paper should be used for filtration. In addition, after filtering the catalyst off it was washed with 1 L of a 1:1 solution of methanol and CHCl_3_, which greatly improves the overall yield. Next, the filtrate was concentrated to give a pale yellow residue, which was dissolved into 5 mL of CH_3_CN and cooled to 0 °C on an ice bath. A solution of CAN (241.8 mg, 0.441 mmol) in 5 mL of H_2_O was added drop wise to the ice-cold CH_3_CN solution and stirred at 0 °C for 15 min. The reaction mixture was allowed to warm to room temperature and stirred for an additional 45 min. The pH of the solution was adjusted with saturated NaHCO_3_ (pH = 8) and extracted with CHCl_3_ (3 × 100 mL). The combined organic extracts were dried over Na_2_SO_4_, filtered and concentrated to afford neo as an orange solid (63.4 mg, 0.202 mmol, 46%). HRMS and ^1^H NMR spectral analysis were in accordance with previously reported values [27,32]. The ^1^H NMR and the HRMS spectra are provided in the Supplementary Information.

## 4. Conclusions

The pyridoacridine compound family has captivated the scientific community because of its interesting and challenging chemistry but also due to its potent biological activities. The development of neo, one of the most successful antitumor pyridoacridines, has been relatively slow due to supply issues despite the report of two total syntheses. In this article, we report an efficient ten-step synthesis of neo with an overall yield of 25%, which is a great improvement over previous syntheses. In this report, we further characterize neo’s potent cytotoxicity in a panel of human tumor cell lines. For solid tumors, the most potent activity was observed against SW48 and SW620 colorectal cancer cells, with the IC_50_ values of 6 and 60 nM, respectively. Neo is significantly more cytotoxic than the conventional TopoIIα poison, etoposide. We show that neo is cytotoxic not cytostatic, and that neo exerts cytotoxicity by inducing G2-M cell cycle arrest and apoptosis. These studies support the continued development of neo as a potential therapeutic for the treatment of cancer. 

## Supplementary Files

Supplementary File 1
