# Supplementary material for: An Improved High Yield Total Synthesis and Cytotoxicity Study of the Marine Alkaloid Neoamphimedine: An ATP-Competitive Inhibitor of Topoisomerase IIα and Potent Anticancer Agent
Source: Mar Drugs. 2014 Sep 19;12(9):4833–50. doi: 10.3390/md12094833 (PMC4178486; doi:10.3390/md12094833)
Supplement: Supplementary File 1 [file marinedrugs-12-04833-s001.pdf]

Supplementary Information

Table of Contents

General methods/materials, and unsuccessful design approaches to prepare neo……………………………………………………………………………S2
Unsuccessful design approaches to prepare neo continued……………………………………………………………………………………S3–S4
^{1}H and ^{13}C NMR for intermediate compounds including expansion/blown up spectra as well as the ^{1}H NMR and HRMS of neoamphimedinepgs…S5–S24

General methods and materials: All reagents were purchased from commercial sources and used as received, unless otherwise indicated. All solvents were dried and distilled using standard protocols. All reactions were carried out under a Nitrogen atmosphere unless otherwise noted. All organic extracts were dried over sodium sulfate. Thin layer chromatography (TLC) was performed using aluminum backed plates coated with 60Å Silica gel F_{254}. Plates were visualized using a UV lamp (λ_{max} = 254 nm) and/or by staining with phosphomolybdic acid solution (20 wt% in ethanol). Column chromatography was carried out using 230–400 mesh 60Å silica Gel. Proton (δ_H) and carbon (δ_X) nuclear magnetic resonances were recorded on a Varian INOVA 500 MHz spectrometer (500 MHz proton, 125.7 MHz carbon). The ^{1}H and ^{13}C assignments were based on gradient enhanced COSY, HMQC, HMBC and DEPT 135. All chemical shifts are recorded in parts per million (ppm), referenced to residual solvent frequencies (^{1}H NMR: Me_4Si = 0, $CDCl_3$ = 7.26, D_2O = 4.79, CD_3OD = 4.87 or 3.31, DMSO-d_6 = 2.50, Acetone-d_6 = 2.05 and ^{13}C NMR: $CDCl_3$ = 77.16; CD_3OD = 49.0, DMSO-d_6 = 39.5, Acetone-d_6 = 29.9). The following splitting abbreviations were used: s = singlet, d = doublet, t = triplet, q = quartet, p = pentet, m = multiplet, br = broad. High-resolution mass spectra (HRMS) were recorded on a Bruker Q-TOF-2 Micromass spectrometer equipped with lock spray, using ESI with methanol as the carrier solvent. Accurate mass measurements were performed using leucine enkephalin as a lock mass and the data were processed using MassLynx 4.1. Exact *m/z* values are reported in Daltons. Melting points (m.p.) were determined using a Stuart SMP40 apparatus and are uncorrected. Infrared (IR) spectra were recorded on a Bruker ALPHA FT-IR fitted with a Platium ATR diamond sampler (examined neat). Absorption maxima (ν_{max}) are recorded in wavenumbers (cm^{-1}).

Unsuccessful design and approaches to prepare neoamphimedine (neo): In our first attempt to optimize the synthesis of neo we investigated whether the formation of the **E**-ring (see Figure 2 in the main article for ring lettering) early in the synthesis would improve the overall yield in fewer steps (Figure S1). By design this approach appeared logical because of the high temperature and strong acid required for ring closure. We reasoned that such harsh conditions may be the cause of the low yield originally reported by LaBarbera *et al.* (reference 32 in the main article) in the final steps of the original neo synthesis. Initially, this reasoning was sound evidenced by the smooth formation of the iso quinolone **E**-ring **S2** from **S1** in 4 steps with an overall yield of 71% (Figure S1). However, attempted thermal cyclization of **S2** with diphenyl ether at various temperatures failed to produce the desired compound **S3**, presumably due to the electronic effect of the isoquinolone **E**-ring of **S2**.

Figure S1. Compound **S2** was prepared in 4 steps with an overall yield of 71% but failed to form **S3** using various thermal cyclizaiton conditions.

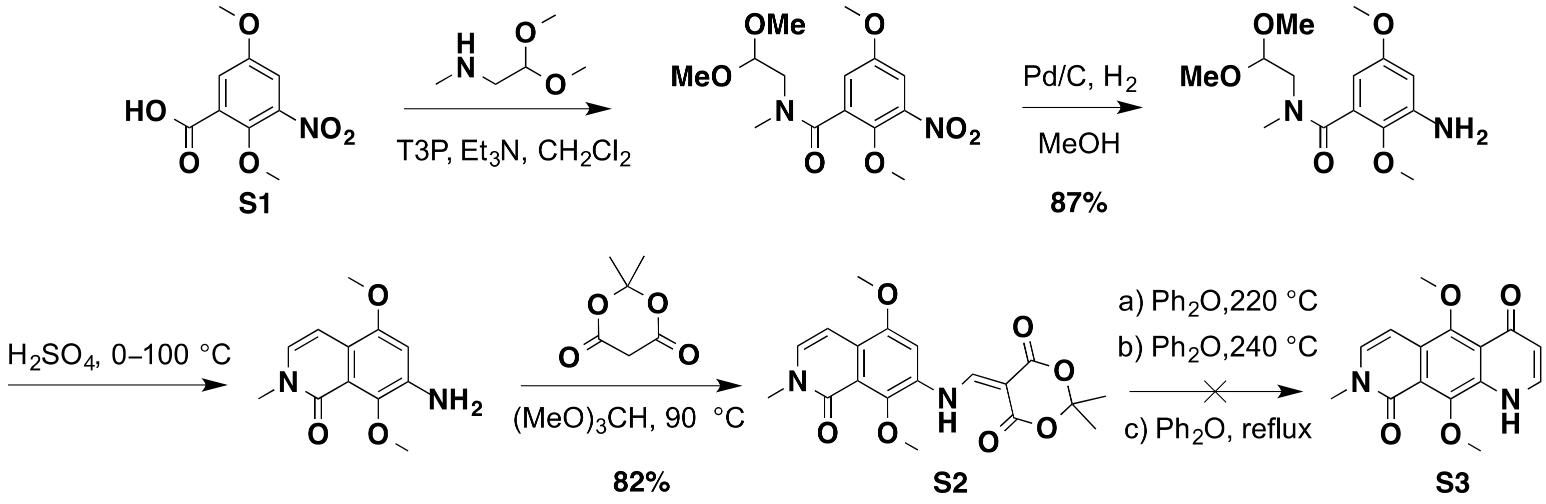

To further investigate this electronic effect we utilized compound **4**, described in the main article in Scheme 2, by derivatization with methylamino acetaldehyde dimethyl acetal. Subsequent acid catalized ring closure failed to provide the desired product containing the **E**, **C**, and **D** rings (figure not shown). Thus, we concluded that this 3-ring structure **S3** containing both the isloquinolone and quinolone ring sytems was not suffieciently stable. Next, we reexamined and designed the synthesis to utilize the well-known Curtius rearrangement and cyclization to synthesize neo (Figure S2). We reasoned that the isocyanate formed from this rearrangement would cyclize readily similar to the Meldrum's derivative under thermal conditions without any strong acid. From compound **S4** we prepared the azide **S5** with overall good yields for each step as shown in Figure S2. Unfortunately, all attempts to generate the **E**-ring via Curtius rearrangement failed. Nevertheless, these tribulations led us to the successful design and completion of the optimized high yielding 10-step synthesis of neoamphimedine as presented in the main article.

Figure S2. Attempted neo synthesis utilizing the thermal Curtius rearrangement.

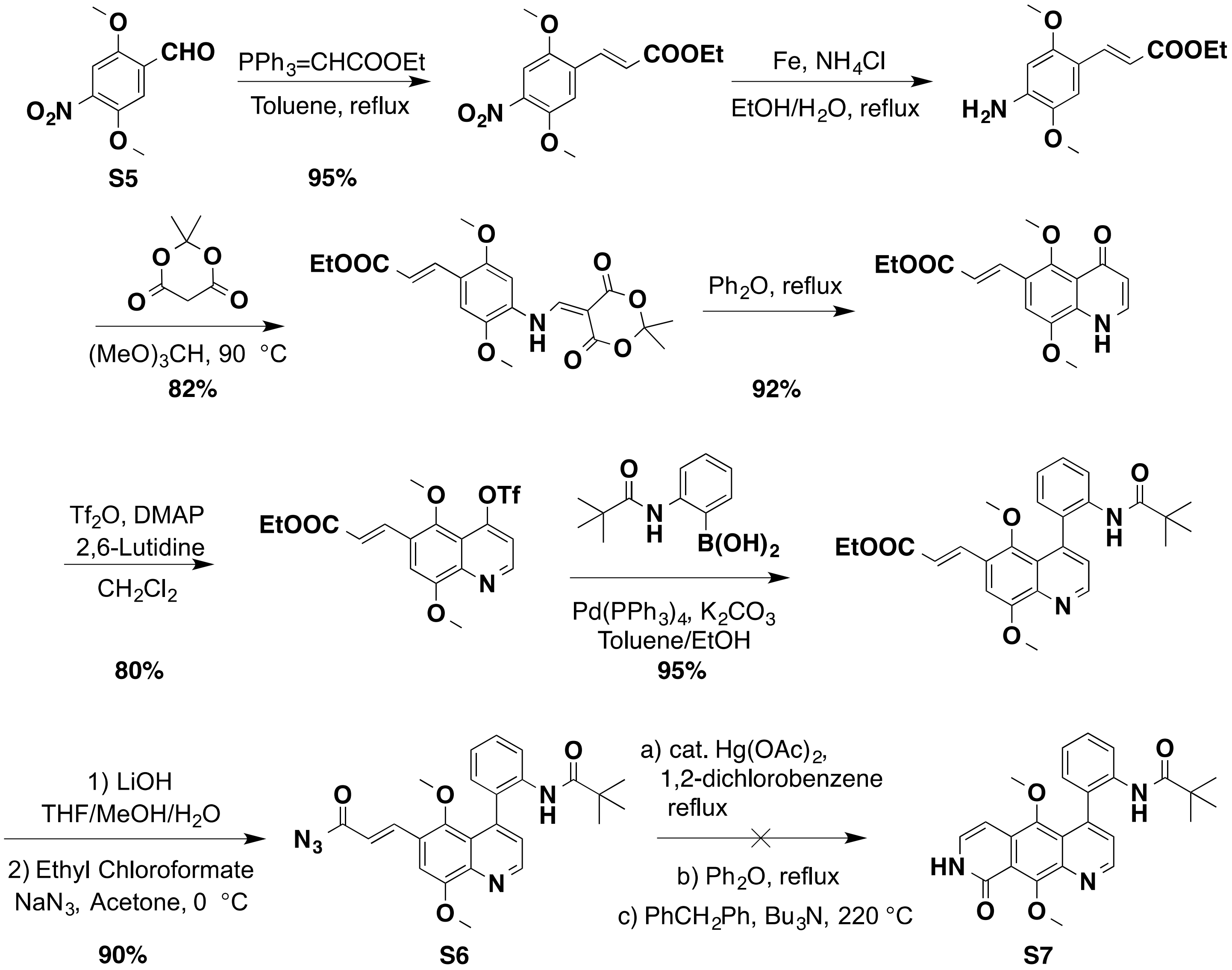

Figure S3. ^{1}H NMR for methyl 3-((2,2-dimethyl-4,6-dioxo-1,3-dioxan-5-ylidene)methyl-amino)-2,5-dimethoxy-benzoate (**3**).

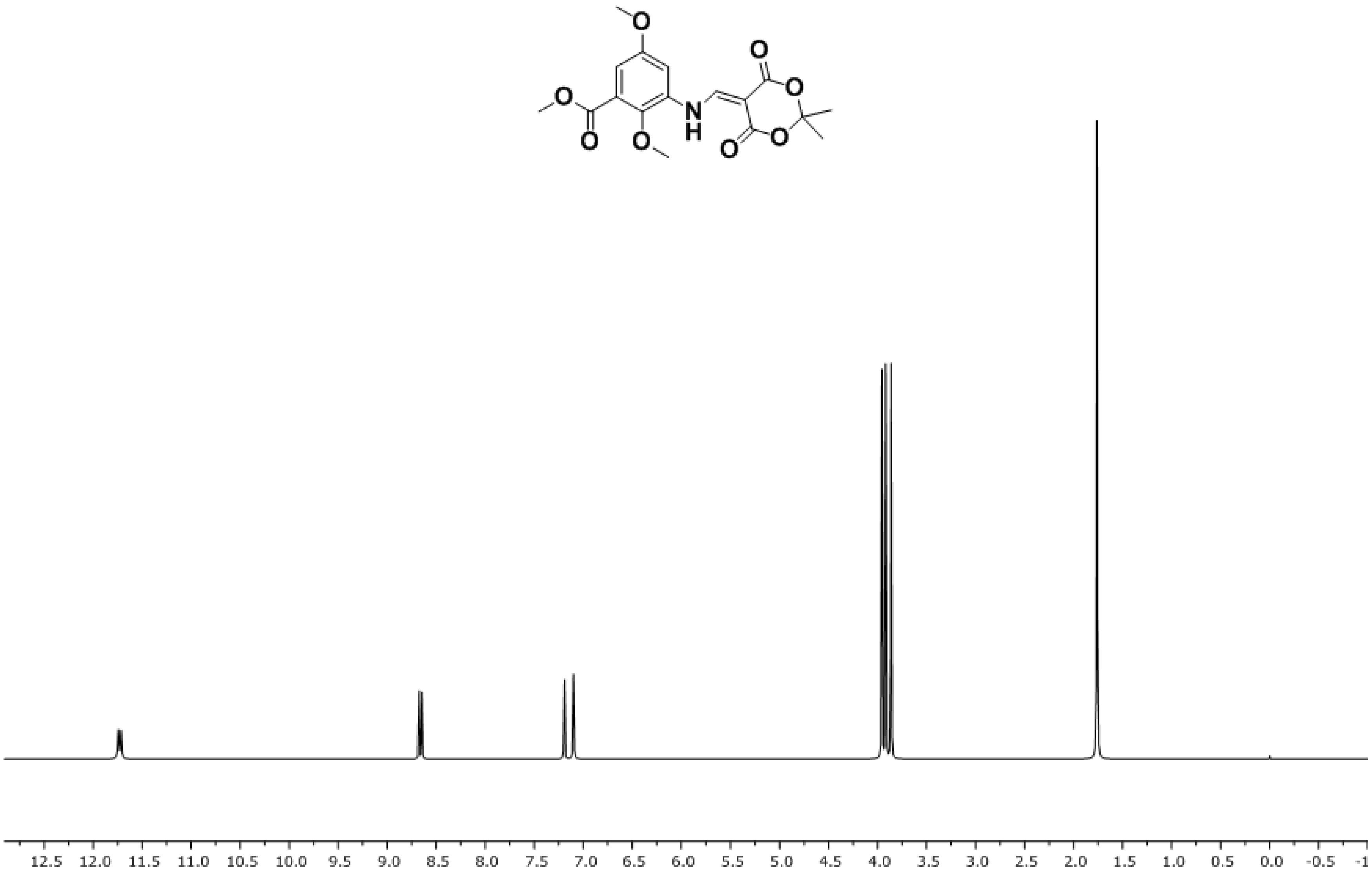

Figure S4. ^{13}C NMR for methyl 3-((2,2-dimethyl-4,6-dioxo-1,3-dioxan-5-ylidene)methyl-amino)-2,5-dimethoxy-benzoate (**3**).

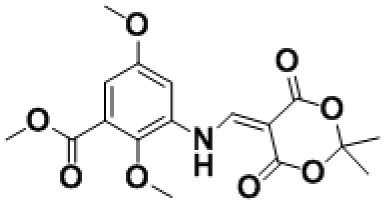

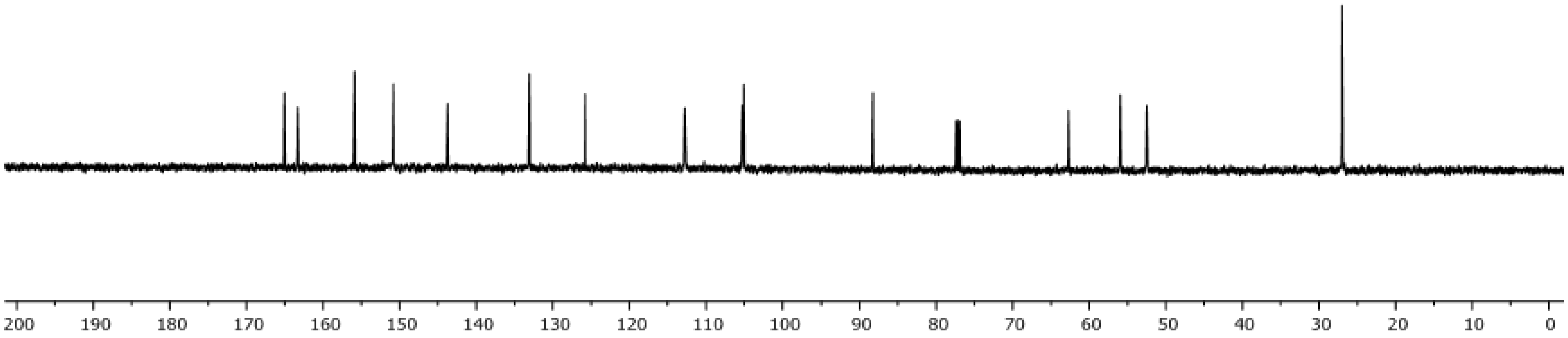

Figure S5. ^{13}C NMR (blown up) for methyl 3-((2,2-dimethyl-4,6-dioxo-1,3-dioxan-5-ylidene)methyl-amino)-2,5-dimethoxy-benzoate (**3**).

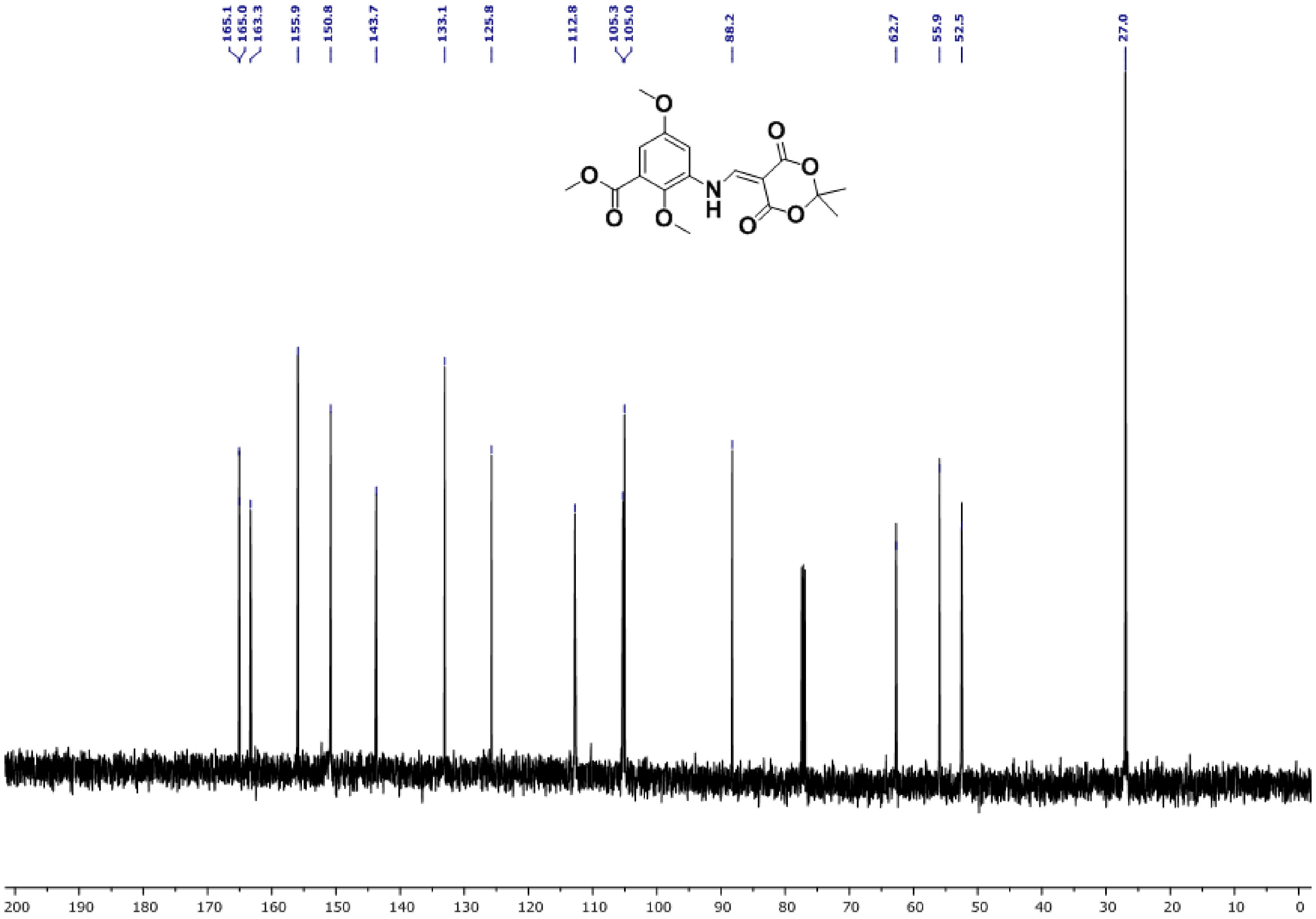

Figure S6. ^{1}H NMR for methyl 1,4-dihydro-5,8-dimethoxy-4-oxo-quinoline-7-carboxylate (**4**).

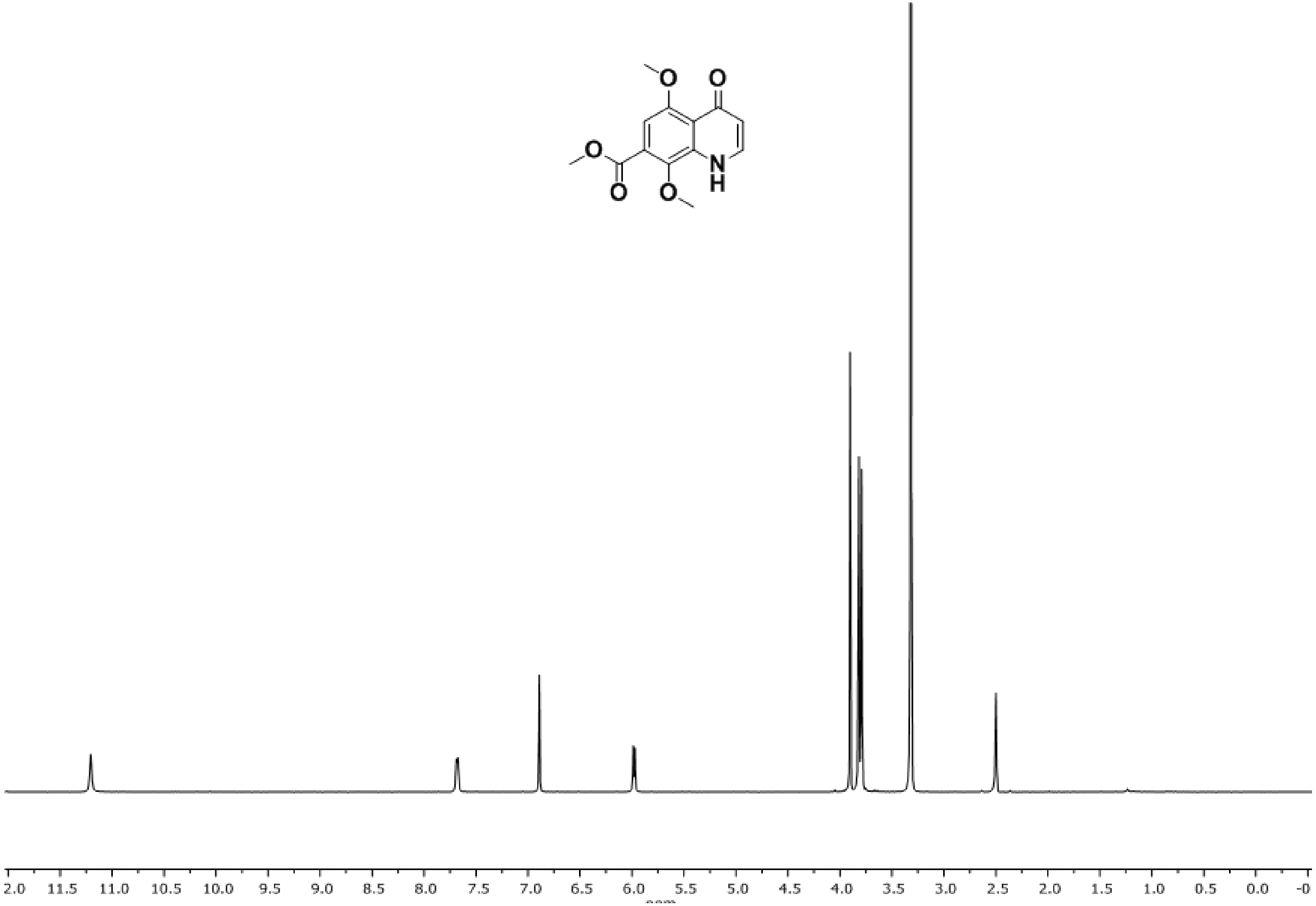

Figure S7. ^{13}C NMR for methyl 1,4-dihydro-5,8-dimethoxy-4-oxo-quinoline-7-carboxylate (**4**).

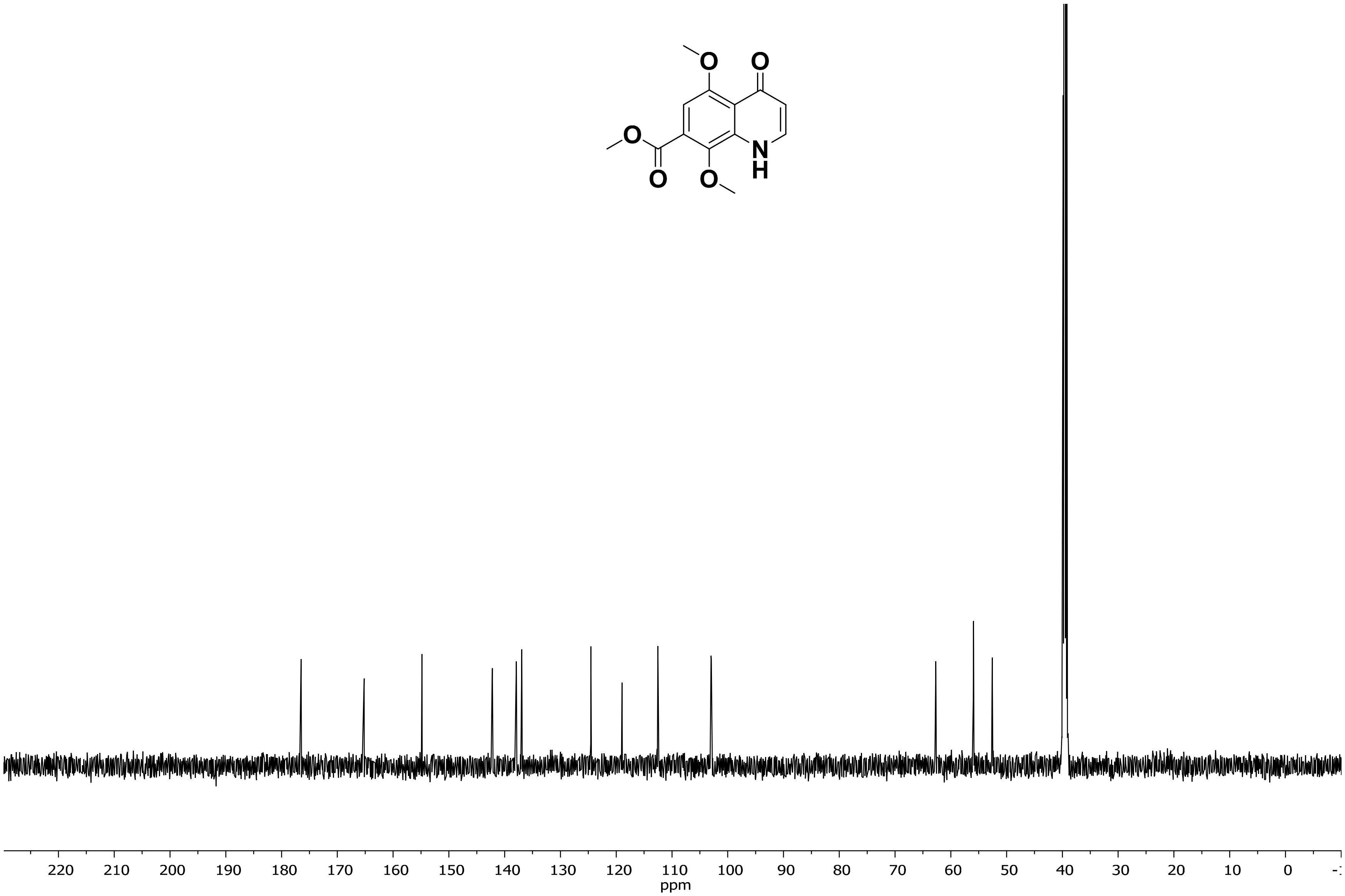

Figure S8. ^{13}C NMR (blown up) for methyl 1,4-dihydro-5,8-dimethoxy-4-oxo-quinoline-7-carboxylate (**4**).

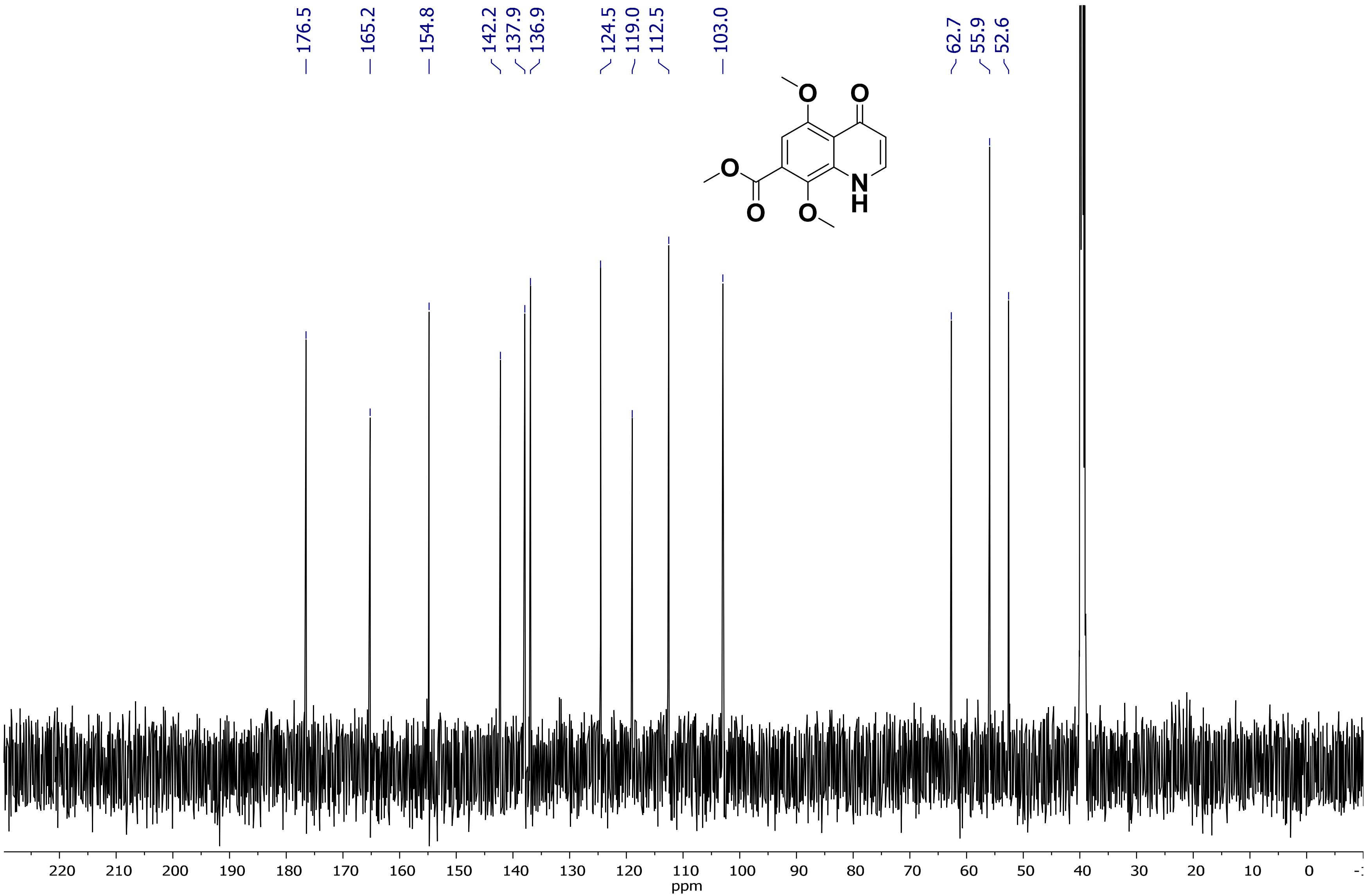

Figure S9. ^{1}H NMR for methyl 5,8-dimethoxy-4-trifluoromethanesulfonyloxy-quinoline-7-carboxylate (**5**).

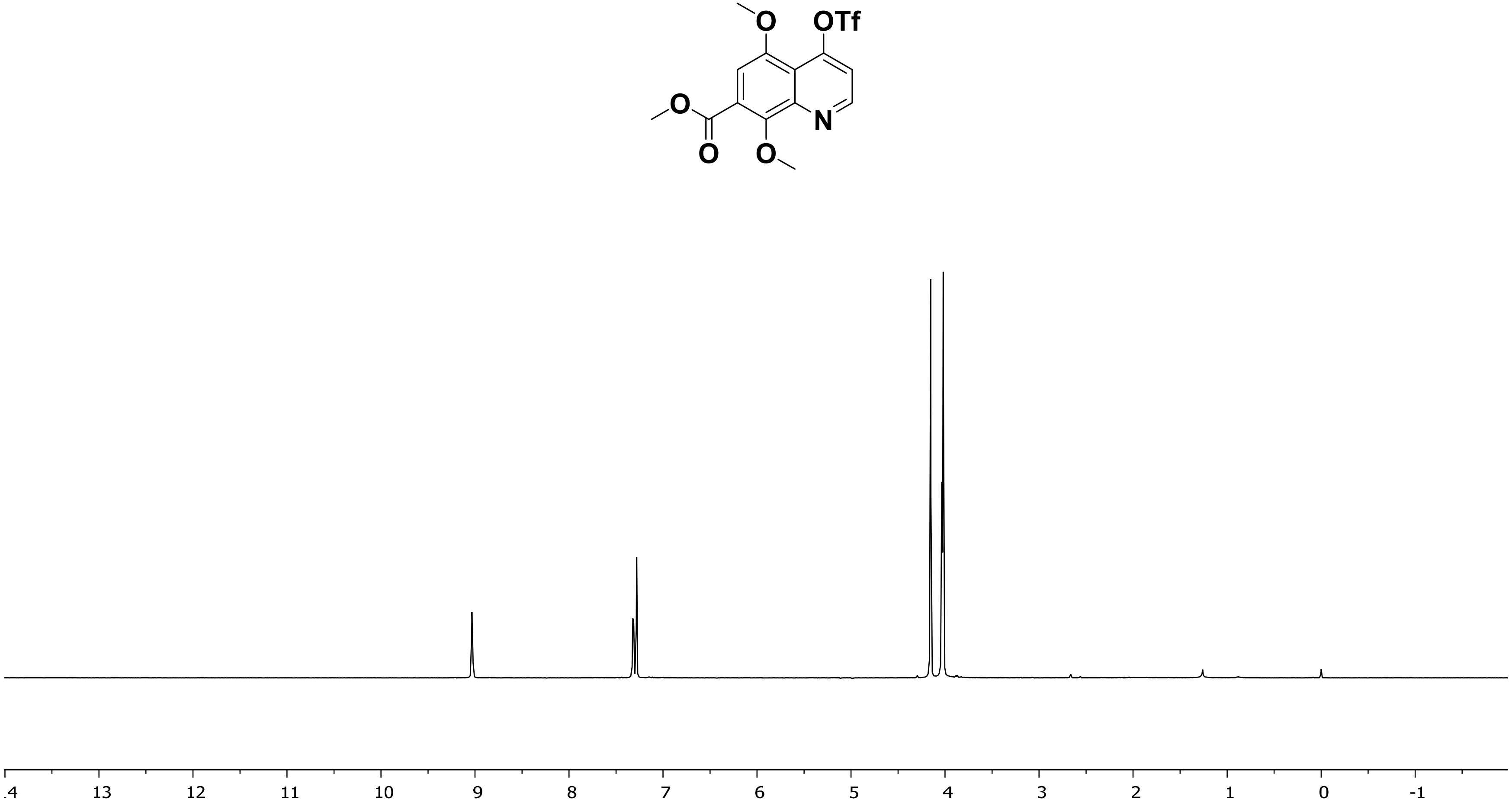

Figure S10. ^{13}C NMR for methyl 5,8-dimethoxy-4-trifluoromethanesulfonyloxy-quinoline-7-carboxylate (**5**).

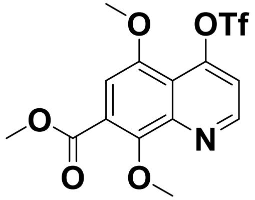

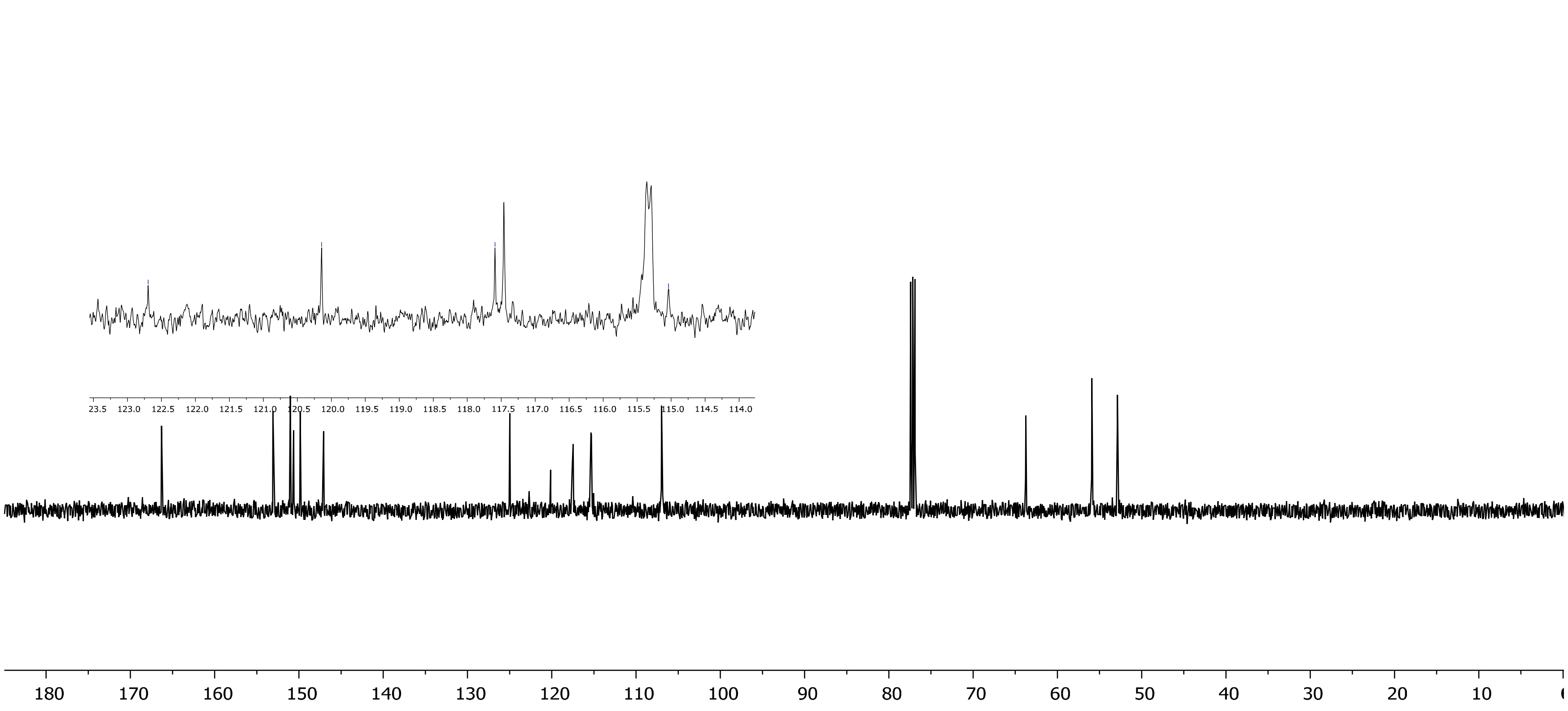

Figure 11. ^{13}C NMR (blown up) for methyl 5,8-dimethoxy-4-trifluoromethanesulfonyloxy-quinoline-7-carboxylate (**5**).

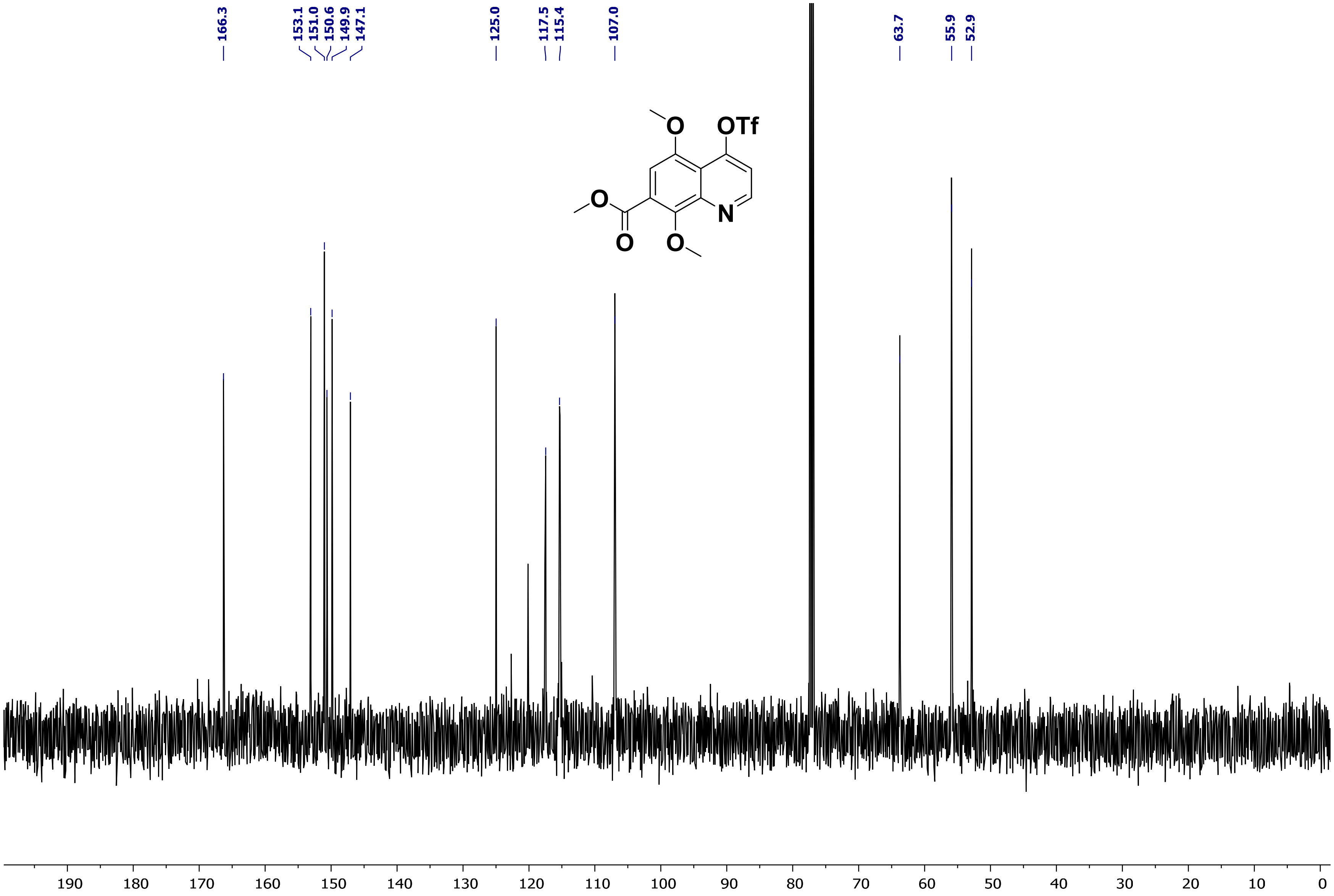

Figure S12. ^{1}H NMR for methyl 4-bromo-5,8-dimethoxy-quinoline-7-carboxylate (**7**).

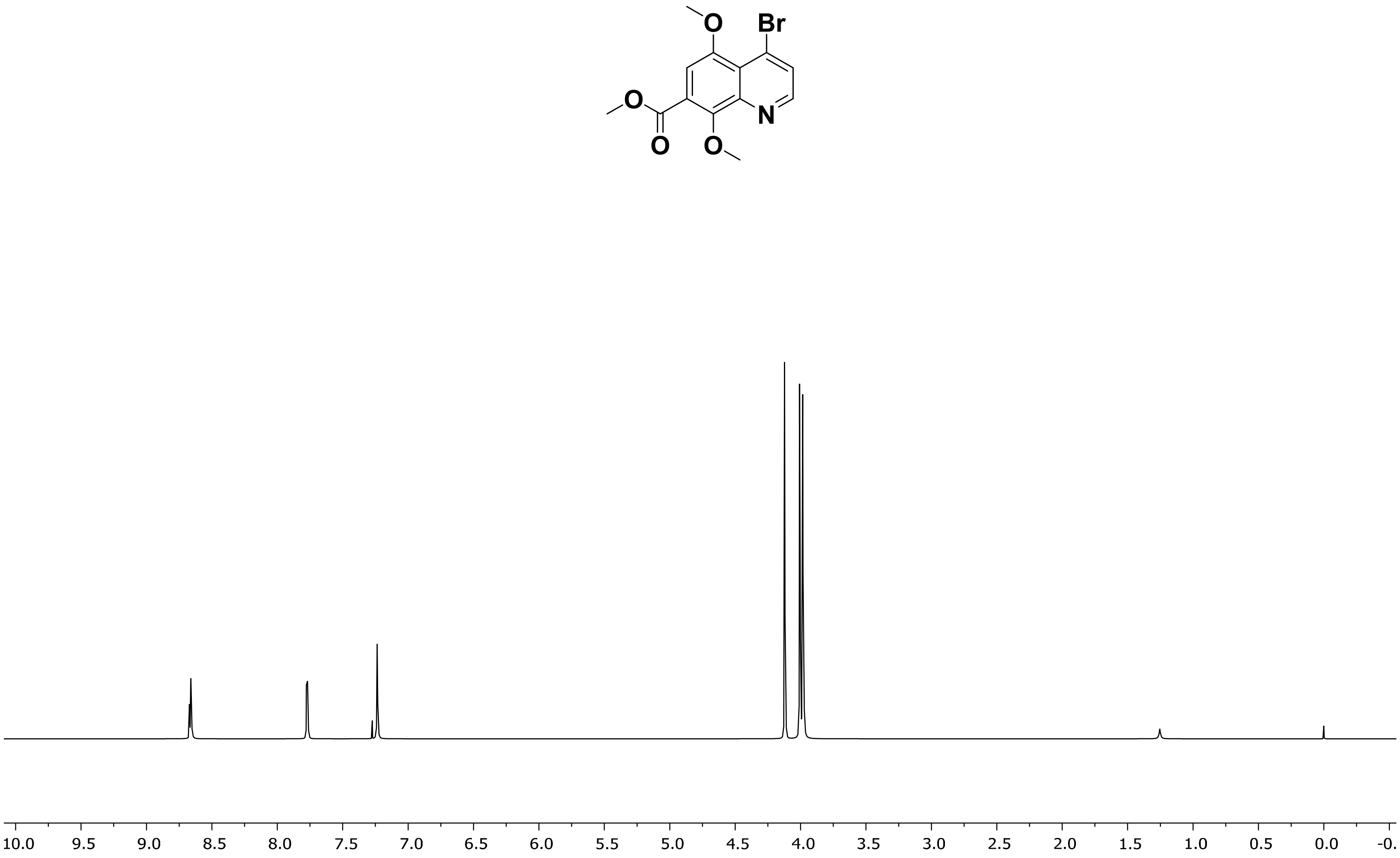

Figure S13. ^{13}C NMR for methyl 4-bromo-5,8-dimethoxy-quinoline-7-carboxylate (**7**).

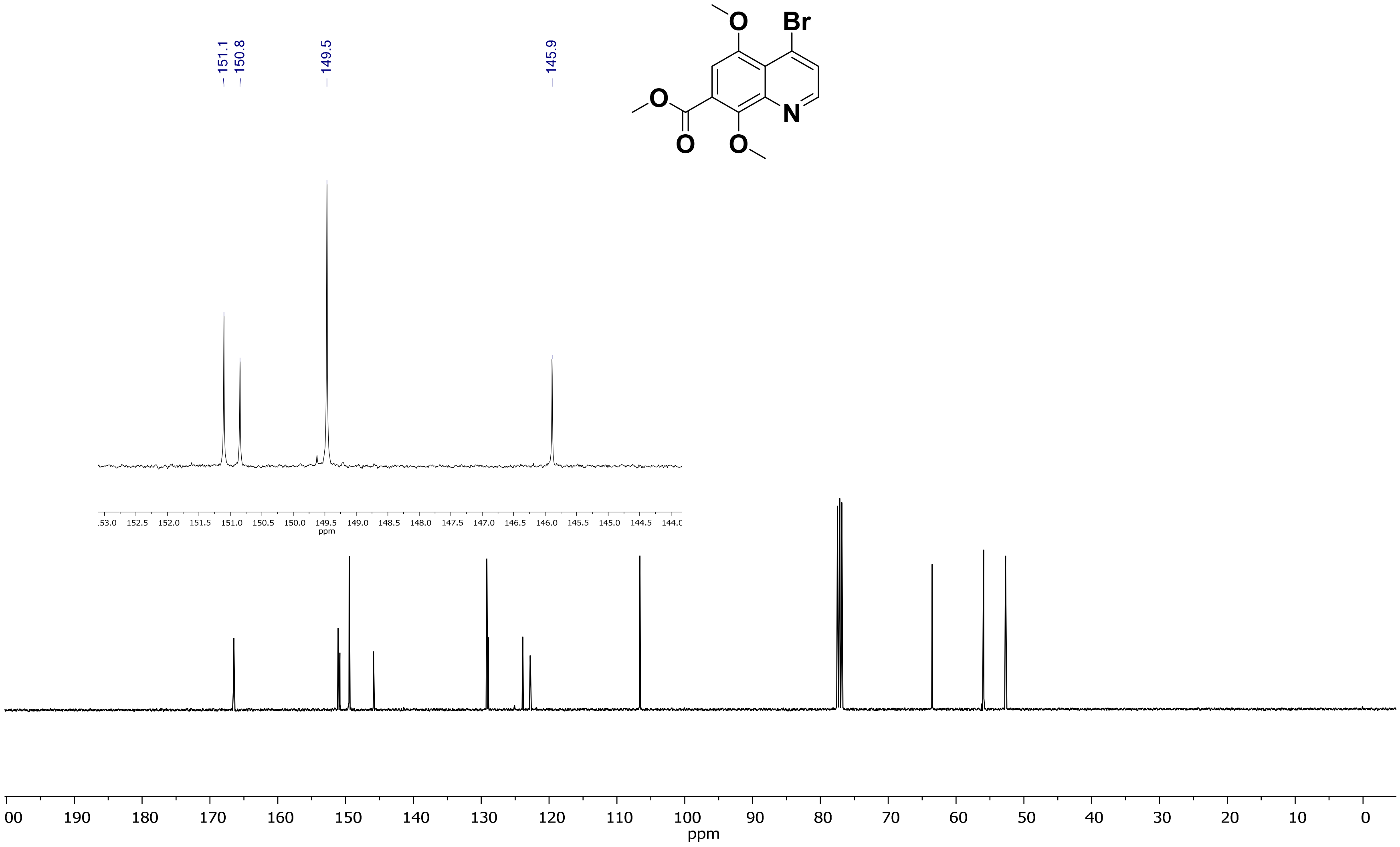

Figure S14. ^{13}C NMR (blown up) for methyl 4-bromo-5,8-dimethoxy-quinoline-7-carboxylate (**7**).

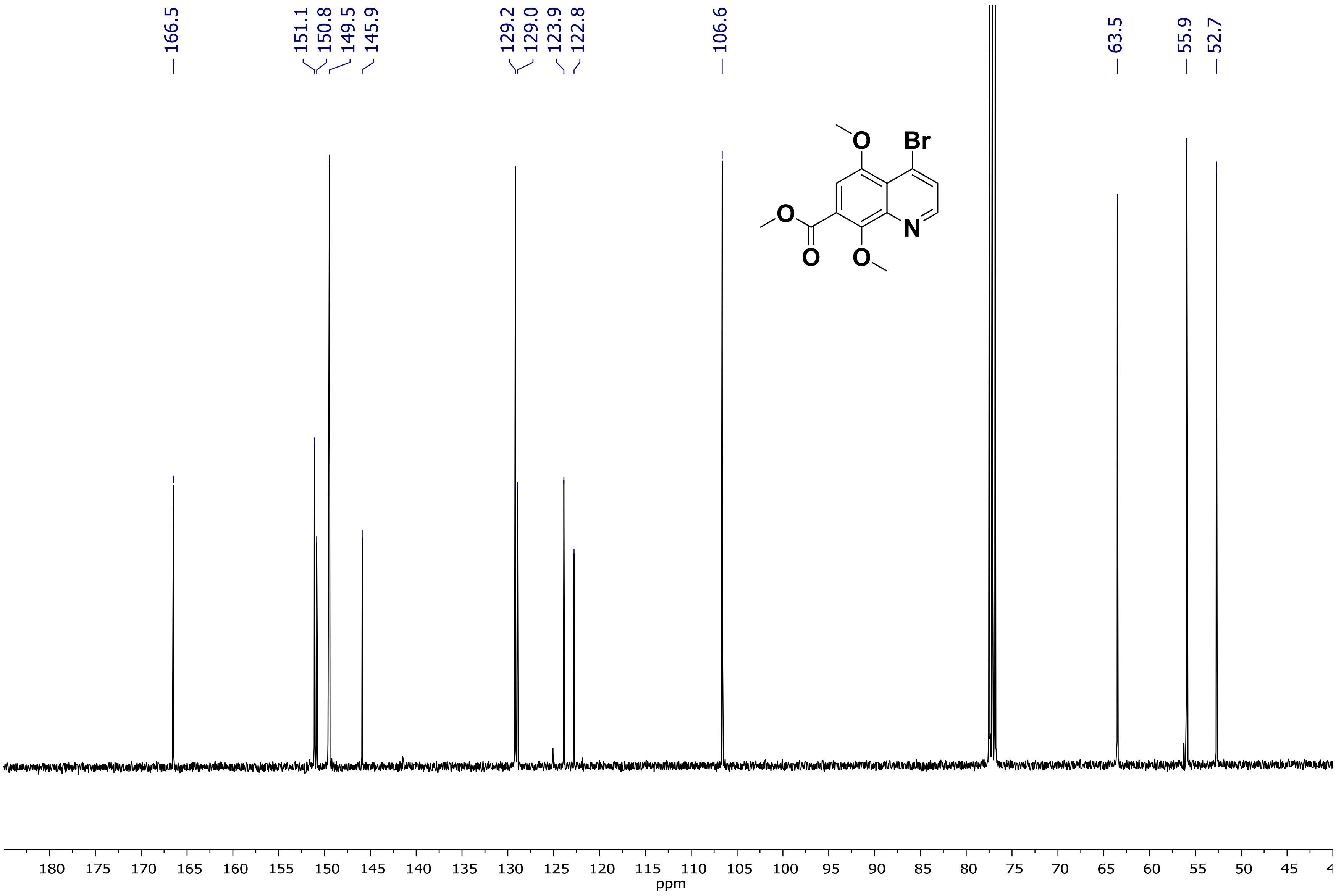

Figure S15. ^{1}H NMR for methyl 5,8-dimethoxy-4-(2-nitrophenyl)-quinoline-7-carboxylate (**6**).

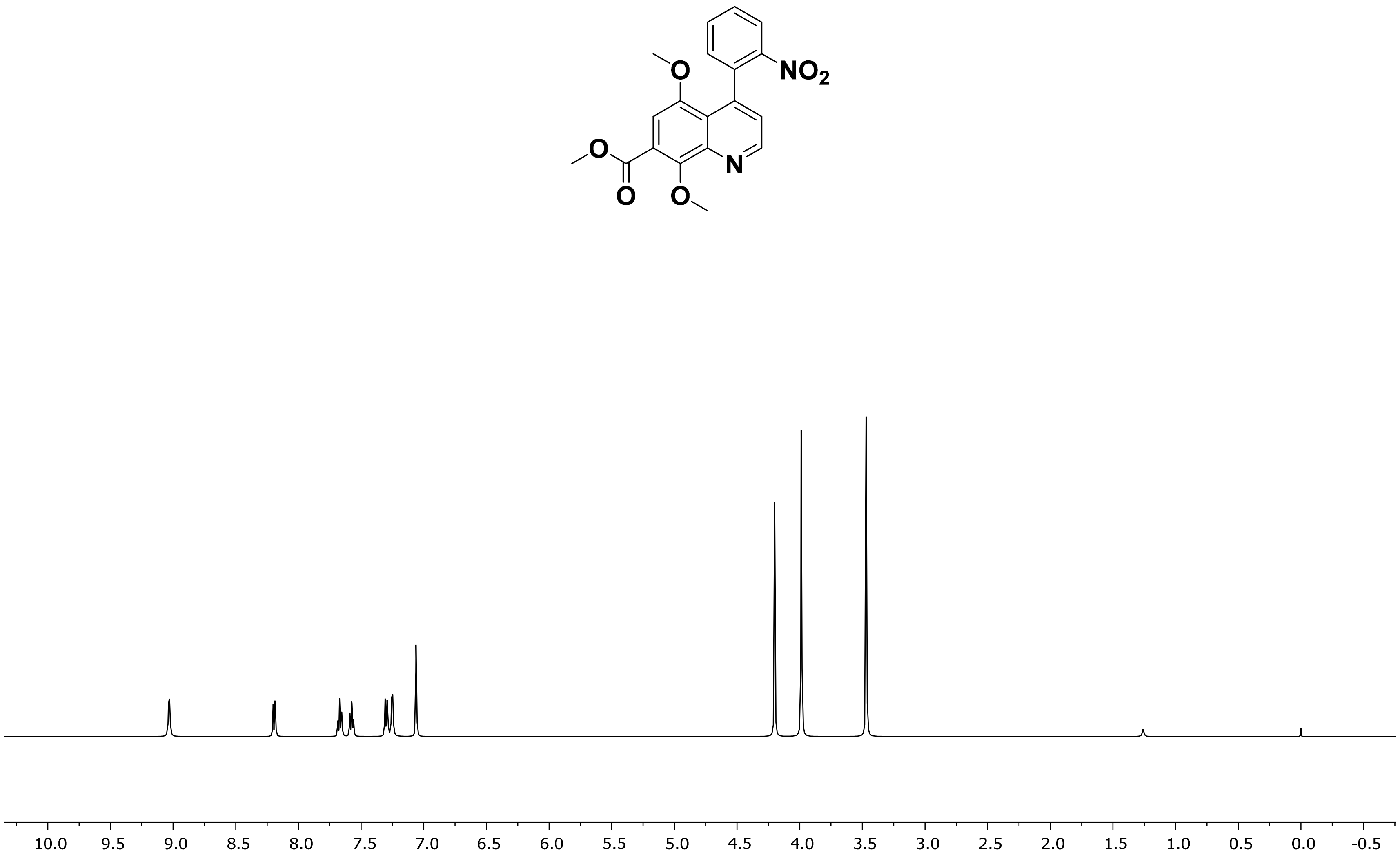

Figure S16. ^{13}C NMR for methyl 5,8-dimethoxy-4- (2-nitrophenyl)-quinoline-7-carboxylate (**6**).

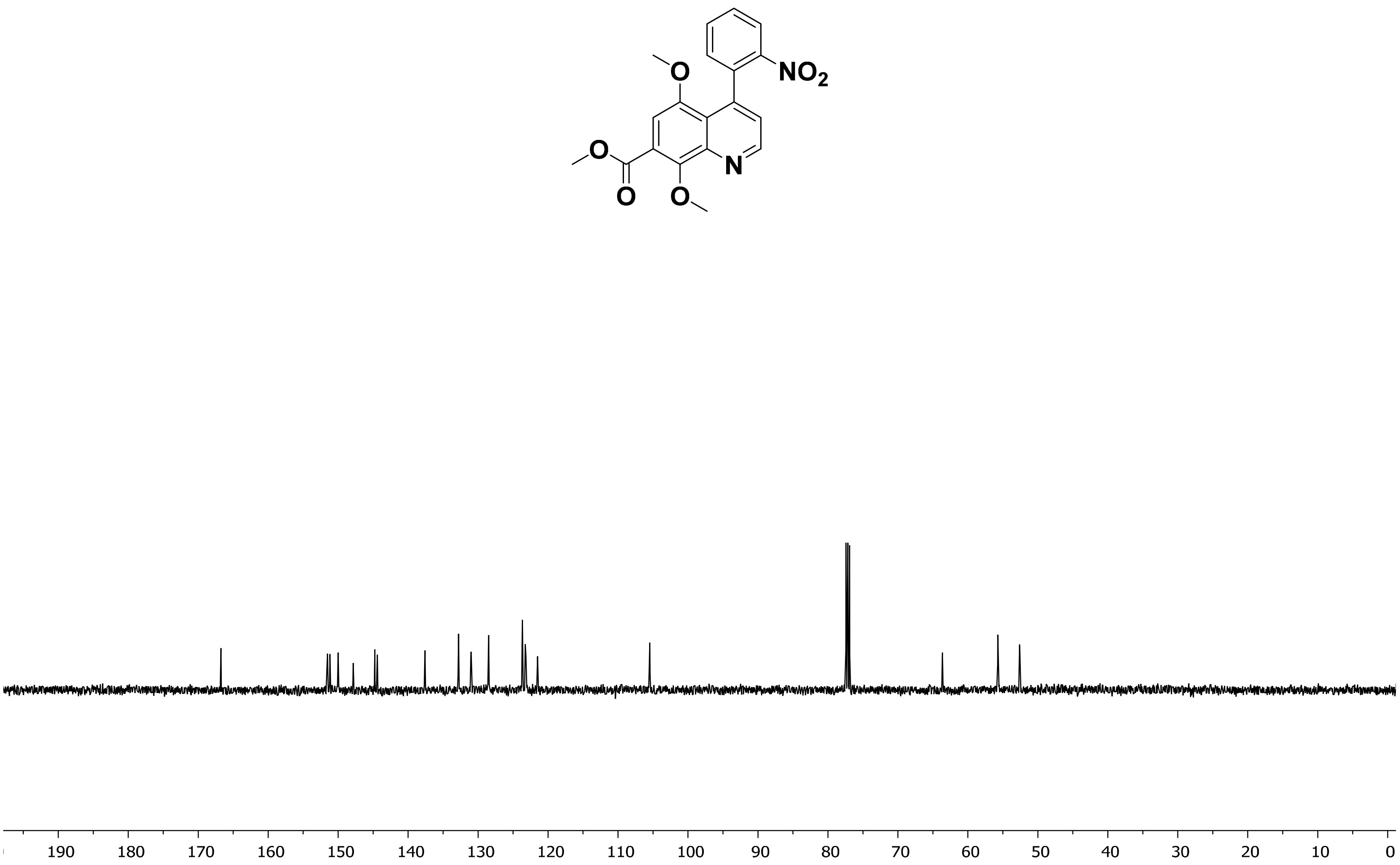

Figure S17. ^{13}C NMR (blown up) for methyl 5,8-dimethoxy-4-(2-nitrophenyl)-quinoline-7-carboxylate (**6**).

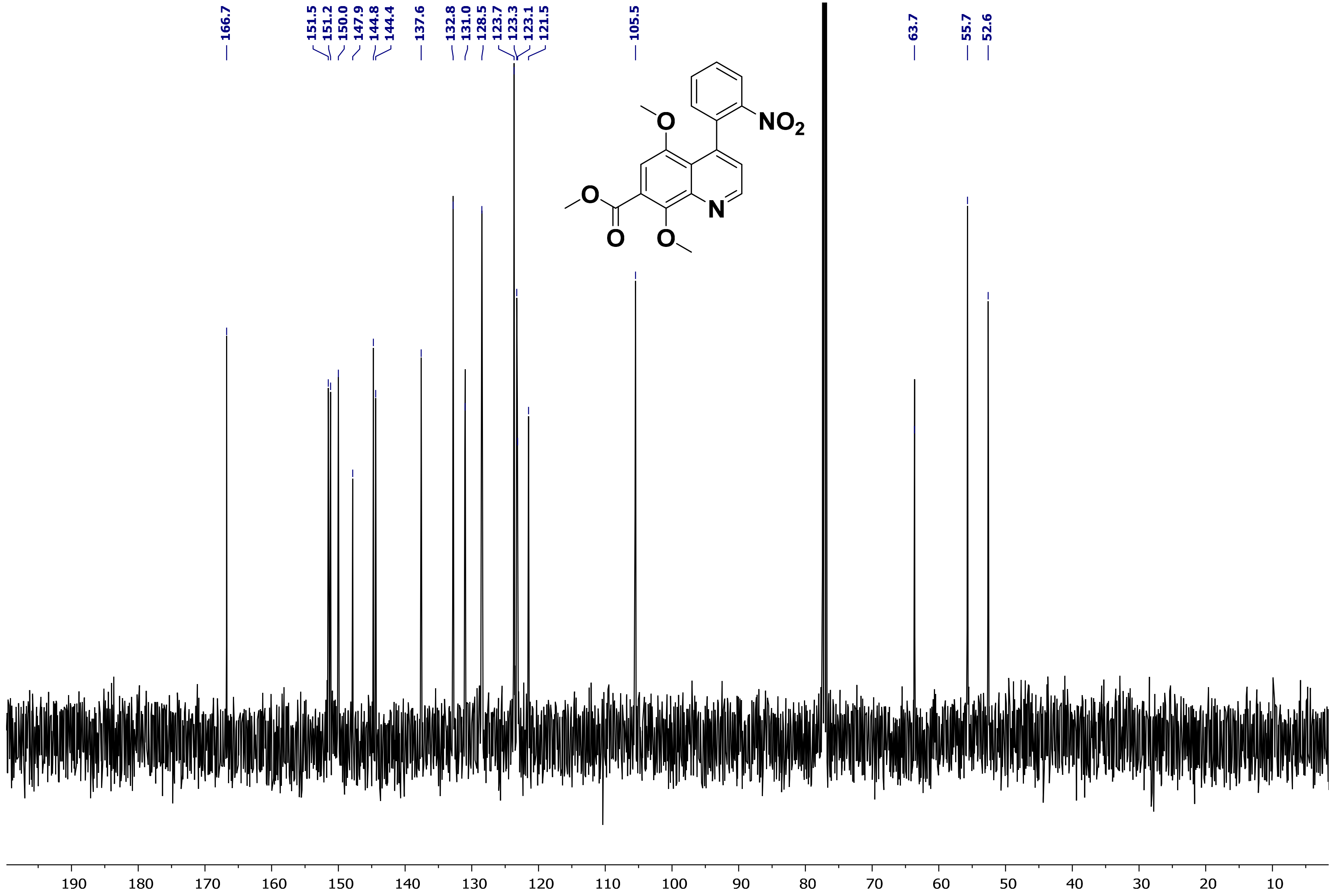

Figure S18. ^{1}H NMR for 8-methyl-4-(2-nitrophenyl)-pyrido-[4,3-*g*]-quinoline-5,9,10-trione.

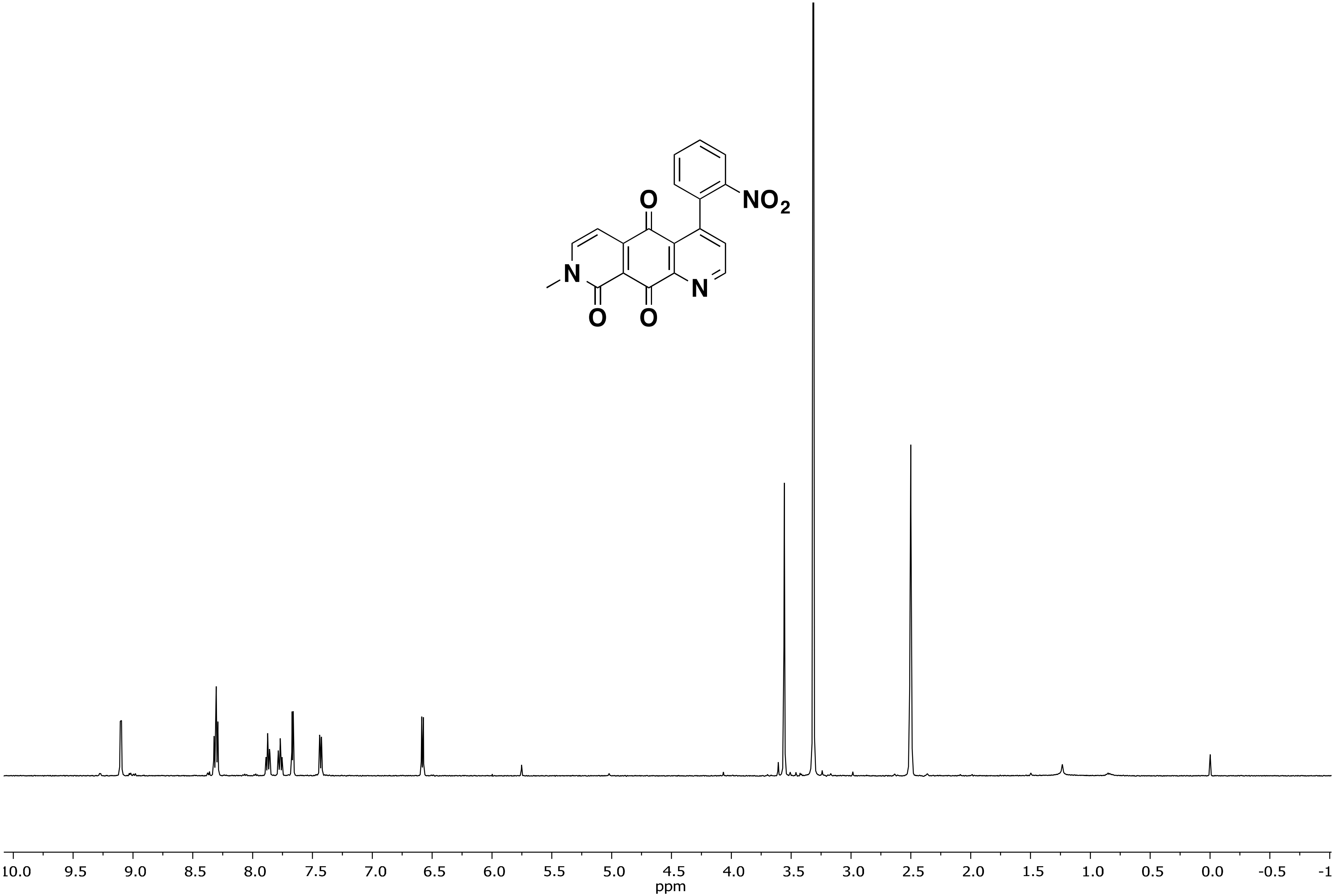

Figure S19. ^{13}C NMR for 8-methyl-4-(2-nitrophenyl)-pyrido-[4,3-*g*]-quinoline-5,9,10-trione.

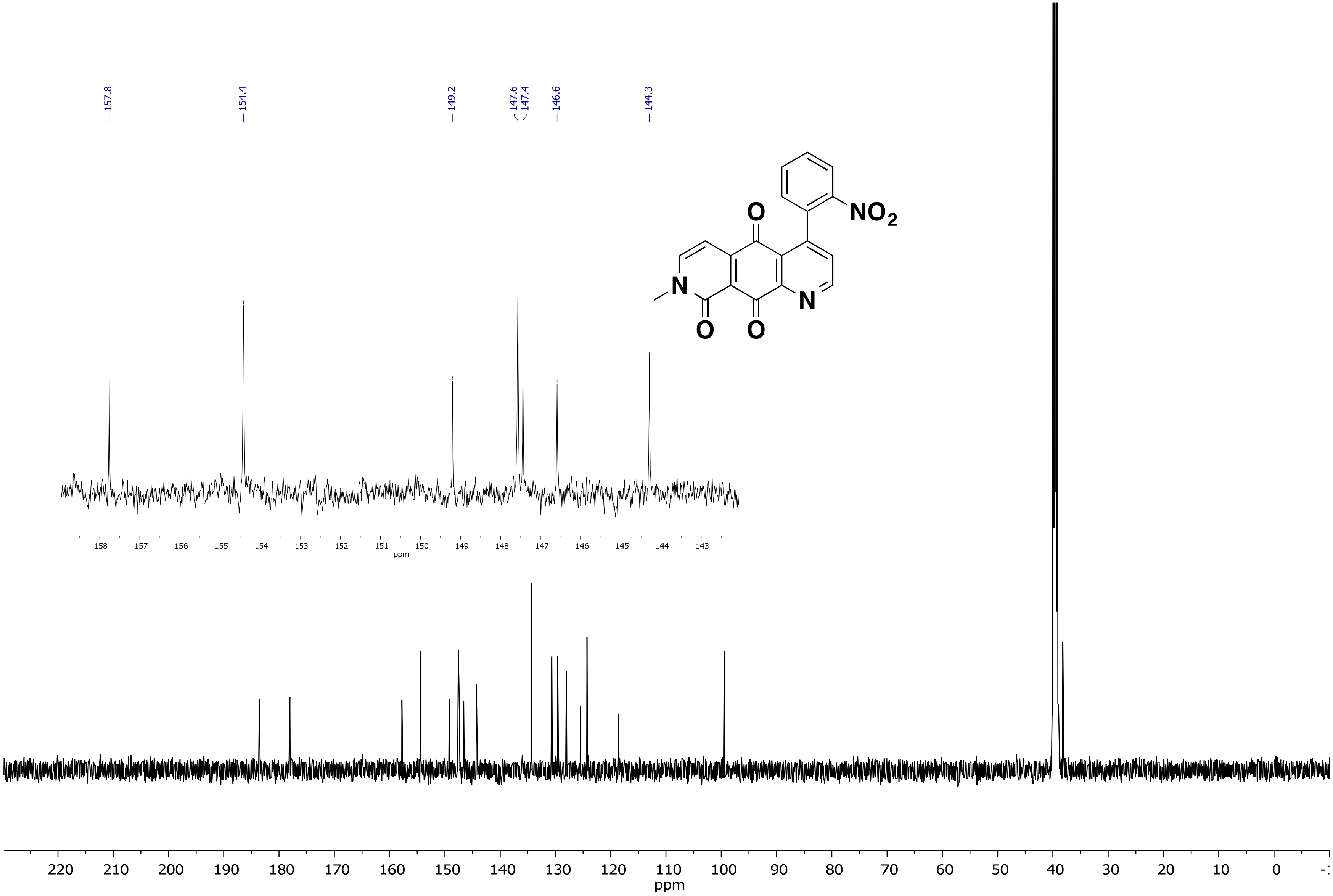

Figure S20. ^{13}C NMR (blown up) for 8-methyl-4-(2-nitrophenyl)-pyrido-[4,3-*g*]-quinoline-5,9,10-trione.

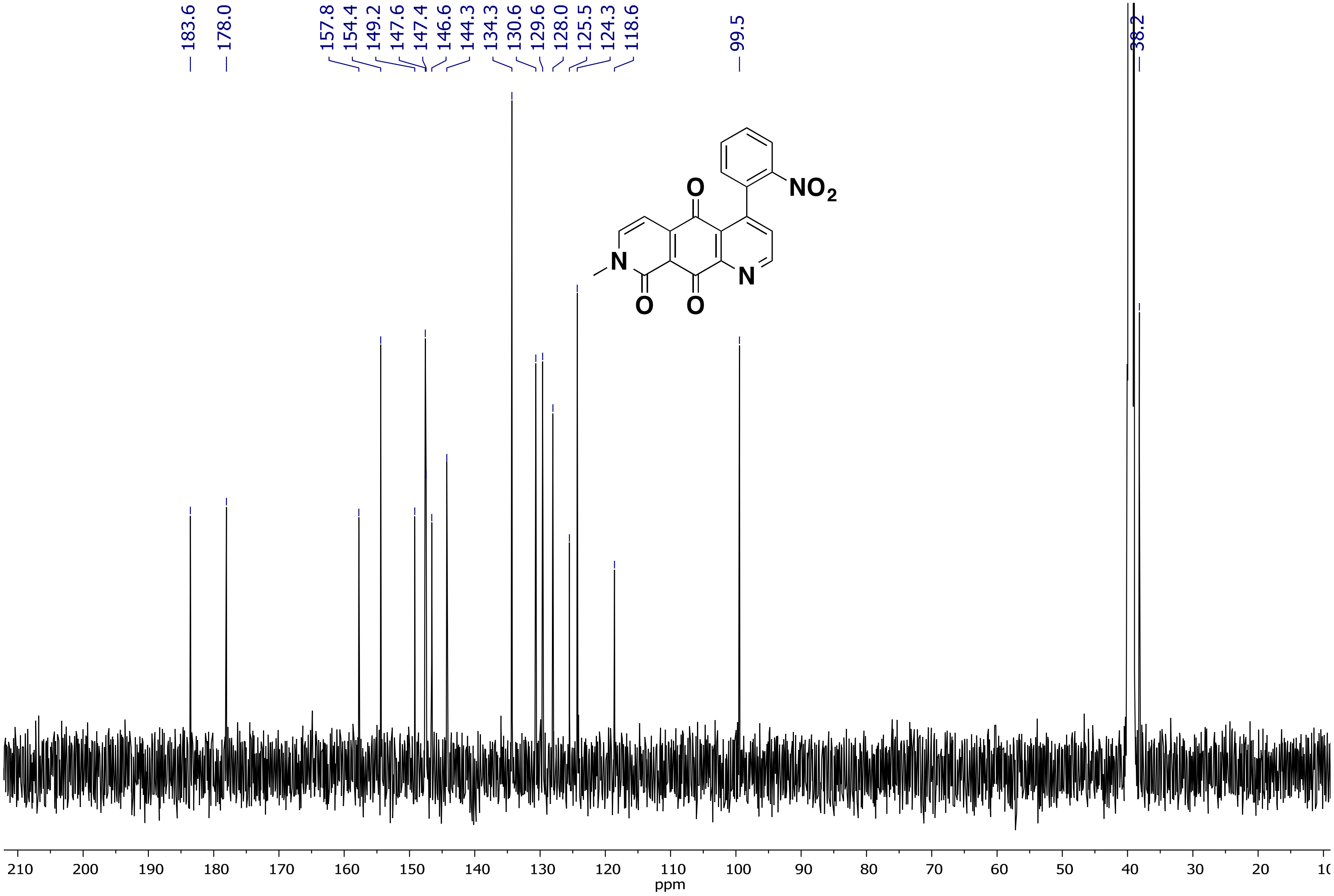

Figure S21. ^{1}H NMR for neoamphimedine.

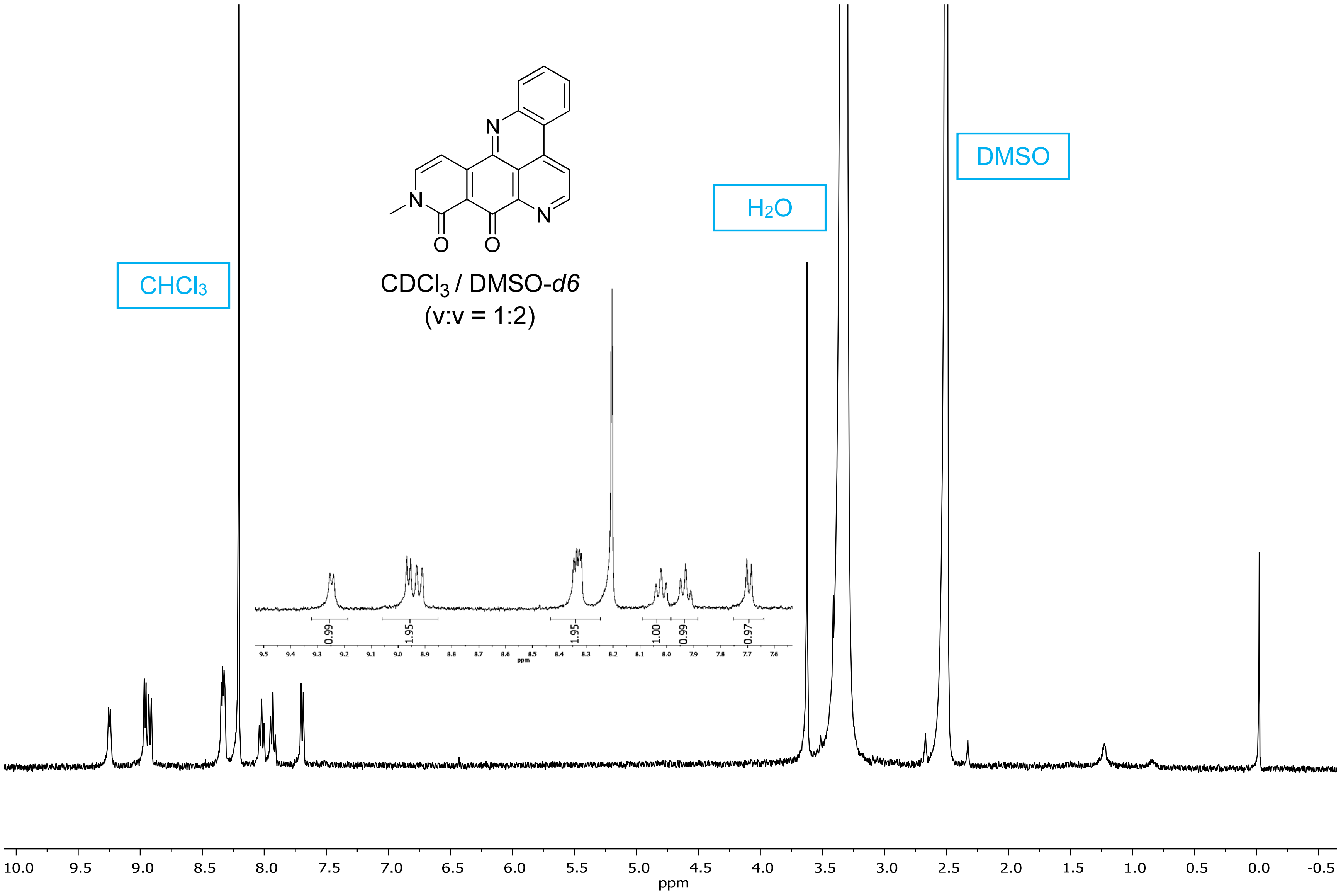

Figure S22. HRMS for neoamphimedine.

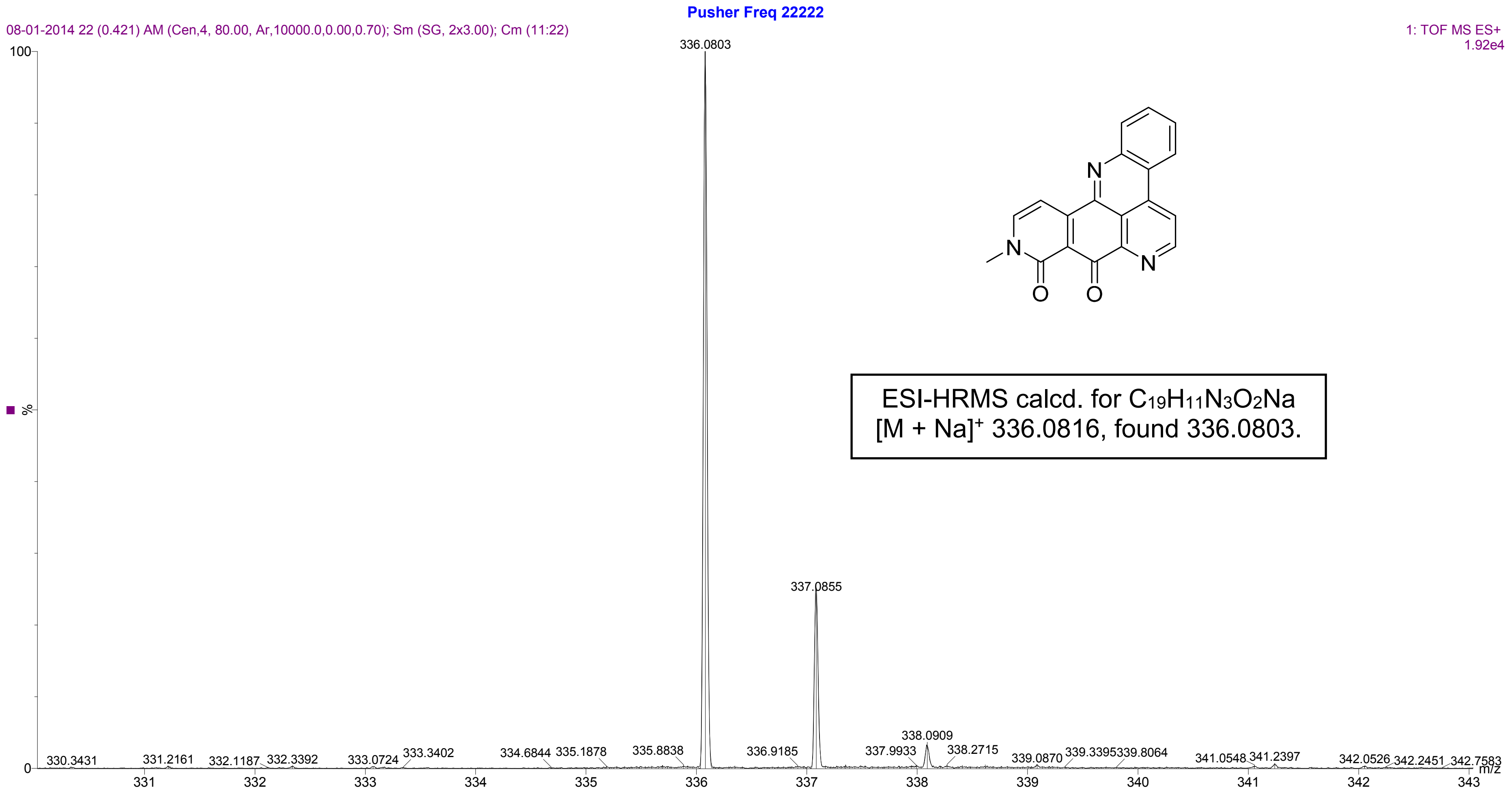

© 2014 by the authors; licensee MDPI, Basel, Switzerland. This article is an open access article distributed under the terms and conditions of the Creative Commons Attribution license (http://creativecommons.org/licenses/by/4.0/).
